# Identification of novel coenzyme Q_10_ biosynthetic proteins Coq11 and Coq12 in *Schizosaccharomyces pombe*

**DOI:** 10.1016/j.jbc.2023.104797

**Published:** 2023-05-06

**Authors:** Ikuhisa Nishida, Yuki Ohmori, Ryota Yanai, Shogo Nishihara, Yasuhiro Matsuo, Tomohiro Kaino, Dai Hirata, Makoto Kawamukai

**Affiliations:** 1 Department of Life Sciences, Faculty of Life and Environmental Sciences, Shimane University, Matsue, Japan; 2 Sakeology Center, Niigata University, Niigata, Japan; 3 Institute of Agricultural and Life Sciences, Academic Assembly, Shimane University, Matsue, Japan

**Keywords:** coenzyme Q_10_, biosynthetic proteins, Coq11, Coq12, *Schizosaccharomyces pombe*, *p*-hydroxybenzoic acid, oxidoreductase

## Abstract

Coenzyme Q (CoQ) is an essential component of the electron transport system in aerobic organisms. CoQ_10_ has ten isoprene units in its quinone structure and is especially valuable as a food supplement. However, the CoQ biosynthetic pathway has not been fully elucidated, including synthesis of the *p*-hydroxybenzoic acid (PHB) precursor to form a quinone backbone. To identify the novel components of CoQ_10_ synthesis, we investigated CoQ_10_ production in 400 *Schizosaccharomyces pombe* gene-deleted strains in which individual mitochondrial proteins were lost. We found that deletion of *coq11* (an *S. cerevisia*e *COQ11* homolog) and a novel gene designated *coq12* lowered CoQ levels to ∼4% of that of the WT strain. Addition of PHB or *p*-hydroxybenzaldehyde restored the CoQ content and growth and lowered hydrogen sulfide production of the *Δcoq12* strain, but these compounds did not affect the *Δcoq11* strain. The primary structure of Coq12 has a flavin reductase motif coupled with an NAD^+^ reductase domain. We determined that purified Coq12 protein from *S. pombe* displayed NAD^+^ reductase activity when incubated with ethanol-extracted substrate of *S. pombe*. Because purified Coq12 from *Escherichia coli* did not exhibit reductase activity under the same conditions, an extra protein is thought to be necessary for its activity. Analysis of Coq12-interacting proteins by LC–MS/MS revealed interactions with other Coq proteins, suggesting formation of a complex. Thus, our analysis indicates that Coq12 is required for PHB synthesis, and it has diverged among species.

Coenzyme Q (CoQ), also called ubiquinone, is a component of the electron transport chain that participates in aerobic respiration in eukaryotes and most prokaryotes (1). The quinone moiety interconverts between the reduced form CoQH_2_ (ubiquinol) and the oxidized form CoQ (ubiquinone), and this property is essential for electron transfer chain function and as a coenzyme for oxidation–reduction enzymes (2). CoQ consists of a benzoquinone ring and a hydrophobic isoprenoid side chain with a certain number of isoprene units in an all-*trans* configuration (3). A CoQ-producing organism produces one type of CoQ as a main product, which is classified according to the length of the isoprenoid side chain (4). For example, *Homo sapiens* and *Schizosaccharomyces pombe* predominantly produce CoQ_10_ with ten isoprene units, whereas *Mus musculus* and *Arabidopsis thaliana* produce CoQ_9_, *Escherichia coli* produces CoQ_8_, and *Saccharomyces cerevisiae* produces CoQ_6_ (5). The side-chain length of CoQ is determined by species-specific polyprenyl diphosphate synthases (PDSs) (6, 7), which utilize isopentenyl diphosphate and farnesyl diphosphate derived from the mevalonate pathway in eukaryotes and archaea, and the methylerythritol phosphate pathway in bacteria and several photosynthetic eukaryotes (3). The main precursor of the benzoquinone ring is *p*-hydroxybenzoic acid (PHB), derived from chorismic acid in prokaryotes and tyrosine in eukaryotes (8). The biosynthetic pathway for the conversion of PHB to CoQ in eukaryotes consists of at least eight steps (Fig. 1). After polyprenyl diphosphate is synthesized by PDS, it is transferred to PHB by PHB–polyprenyl diphosphate transferase (Coq2 or Ppt1). The six-membered ring of prenylated PHB is then modified by three hydroxylations catalyzed by Coq6, Coq7, and a still-unidentified enzyme(s), two *O*-methylations by Coq3, *C*-methylation by Coq5, and decarboxylation by an unknown enzyme(s) (8). In eukaryotes, this pathway has been most comprehensively studied in *S. cerevisiae* (9) and *S. pombe* (10), and knowledge from these yeasts has been extended to several animals and plants (11, 12). The nine genes (*COQ1*–*COQ9*) in *S. cerevisiae* (13) and the ten genes (*dps1*, *dlp1*, *ppt1* [*coq2*], and *coq3–coq9*) in *S. pombe* are absolutely required for CoQ biosynthesis (2, 14, 15, 16). The side-chain determining enzyme is a homomer of Coq1 in *S. cerevisiae* and a heteromer of Dps1 and Dlp1 in *S. pombe* (17). It is a heteromer of PDSS1 and PDSS2 in *H. sapiens* (18) like in *S. pombe*. In addition to these essential genes for CoQ biosynthesis, *COQ10* and *COQ11* are known to participate in CoQ biosynthesis in *S. cerevisiae* (13, 19, 20). Coq10 is a CoQ-binding protein that is moderately involved in CoQ biosynthesis (19, 20), and Coq11 is thought to cooperate with Coq10 (21). Importantly, except for *COQ11*, yeast *COQ* homologous genes are all present in humans (10, 13). However, the functions of *COQ4*, *COQ8*, *COQ9*, and *COQ11* remain to be elucidated (22, 23), and the pathway upstream of PHB synthesis remains poorly understood (24, 25). PHB synthesis involves oxidation of *p*-hydroxybenzaldehyde (PHBALD) catalyzed by Hfd1 in *S. cerevisiae*, and this step is presumed to be catalyzed by its ortholog ALDH3A1 in humans (24).

**Figure 1:**
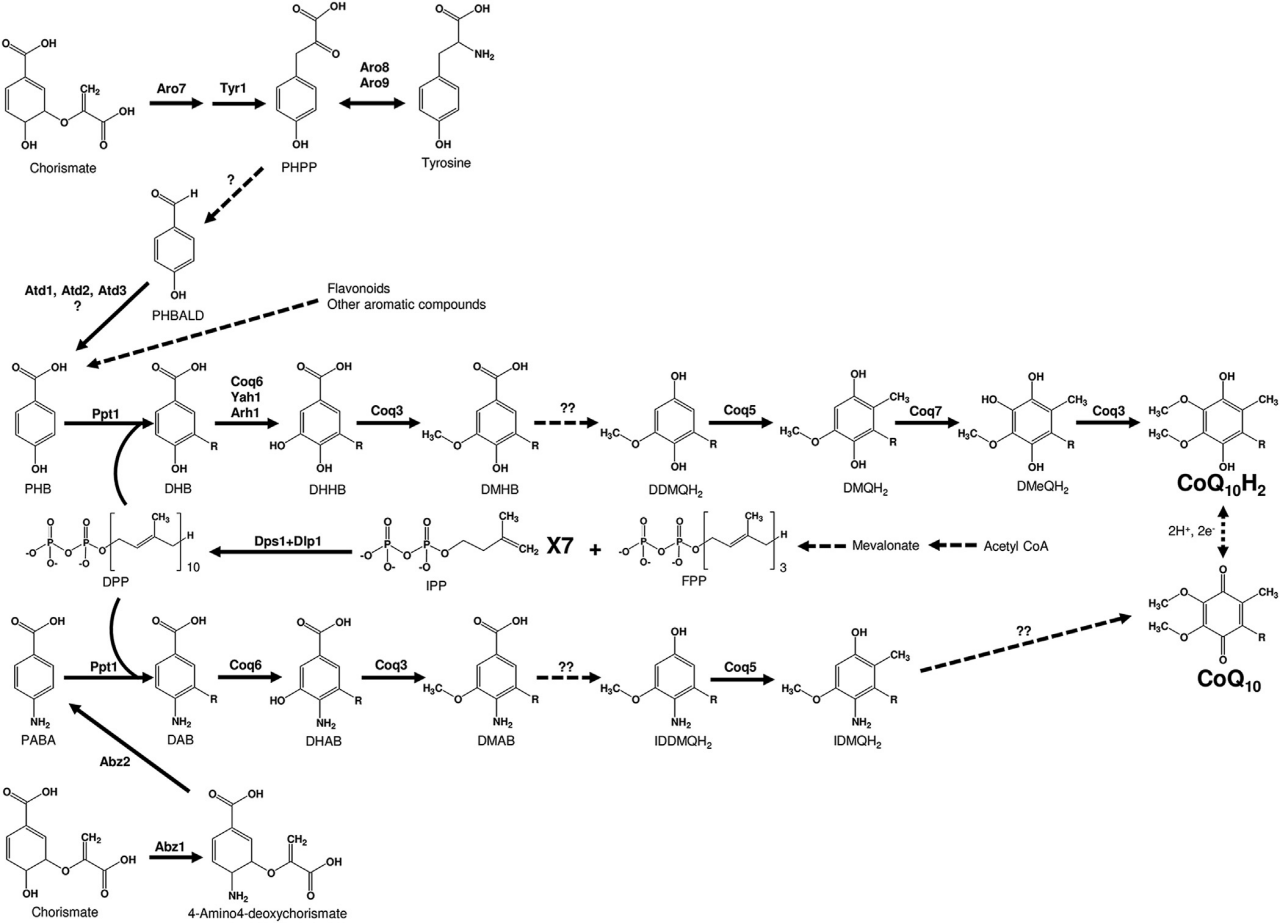
**Overview of the CoQ biosynthetic pathway in *Schizosaccharomyces pombe*.** This figure illustrates the arrangement of CoQ biosynthetic and related proteins in the fission yeast and modified from the figure reported by Awad *et al.* (11). The CoQ biosynthetic pathway has been shown to involve at least 16 nuclear-encoded proteins that are necessary for mitochondrial CoQ biosynthesis in *S. pombe*. *Black dotted arrows* denote more than one step. *Solid arrows* denote a single step attributed to the corresponding yeast polypeptide named above each *arrow*. Yeasts synthesize *p*-hydroxybenzoic acid (PHB) or *p*-aminobenzoic acid (PABA) by *de novo* from chorismate. PHB may also be formed by the metabolism of tyrosine. *S. pombe* cells produce isopentenyl pyrophosphate (IPP) and dimethylally pyrophosphate (DMAPP) as precursors to form farnesyl diphosphate (FPP; n = 3). Decaprenyl diphosphate (DPP; n = 10) is synthesized from FPP and IPP *via* Dps1 + Dlp1 in the fission yeast. *S. pombe* Coq2/Ppt1 attach the polyisoprenyl tail to PHB or PABA. Subsequent to this step, the next three intermediates are identified as yeast decaprenyl intermediates: DHB, 3-decaprenyl-PHB; DHHB, 3-decaprenyl-4,5-dihydroxybenzoic acid; DMHB, 3-decaprenyl-4-hydroxy-5-methoxybenzoic acid. The next three intermediates are hydroquinones: DDMQH_2_, 2-decaprenyl-6-methoxy-1,4-benzenediol; DMeQH_2_, 2-decaprenyl-3-methyl-6-methoxy-1,4,5-benzenetriol; DMQH_2_, 2-decaprenyl-3-methyl-6-methoxy-1,4-benzenediol; to ultimately produce the final reduced product (CoQ_10_H_2_). Coq6 may require ferredoxin Yah1 (Etp1) and ferredoxin reductase Arh1 as in *S. cerevisiae*. It has been shown that PABA as an alternate ring precursor utilized by *S. pombe*(and it is suggested in humans). The next three intermediates are identified as yeast decaprenyl intermediates: DAB, 4-amino-3-decaprenylbenzoic acid; DHAB, 4-amino-3-decaprenyl-5-hydroxybenzoic acid; DMAB, 4-amino-3-decaprenyl-5-methoxybenzoic acid. The next two intermediates are IDDMQH_2_, 4-amino-3-decaprenyl-5-methoxyphenol and IDMQH_2_, 4-amino-3-decaprenyl-2-methyl-5-methoxyphenol. Unknown deamination step is involved in the PABA pathway. Interconversion of (CoQnH_2_) and (CoQn) is shown *via* a reversible two-electron reduction and oxidation. Steps indicated by “?” are catalyzed by unknown enzyme(s).

Studies using stable isotope–labeled *p*-aminobenzoic acid (PABA) and PHB revealed that they are incorporated into the quinone ring of CoQ_6_ in *S. cerevisiae* and CoQ_10_ in *S. pombe* (26, 27, 28). At least in these two different genera of yeasts, in addition to PHB, PABA is utilized as a precursor for CoQ synthesis, but it is not clear how widely PABA is utilized in other species.

In this study, to better understand how biosynthesis of CoQ is controlled, we selected 400 individual gene deletion strains of *S. pombe* in which individual mitochondrial targeting proteins are lost, and explored how these genes affect the synthesis of CoQ_10_. We found that *coq11* (*S. cerevisiae COQ11* ortholog) and the novel gene *coq12* are required for *S. pombe* CoQ_10_ synthesis and proposed that Coq12 is involved in the PHB synthetic pathway.

## Results

### Identification of the novel CoQ_10_ biosynthetic gene in *S. pombe*

To identify novel genes responsible for CoQ_10_ biosynthesis in *S. pombe*, we selected 400 gene disruptants in which individual mitochondrial targeting proteins were lost and analyzed the amount of CoQ_10_. For this purpose, we used the Genome-wide Deletion Mutant Library supplied by Bioneer Corp (29). As a result, we obtained 42 strains in which the CoQ_10_ content was lower than half that of the WT strain. The ten mutants with the lowest CoQ_10_ levels are listed in Table 1. These genes encode oxidoreductases, respiratory chain–related proteins, and mitochondrial porins, which are potential regulators of CoQ biosynthesis. Among them, *S. pombe coq11* (*SPCC1840.09*) is an ortholog of *S. cerevisiae COQ11*, which is required for CoQ biosynthesis (21), but its role in *S. pombe* has not been analyzed. Our observation that the *S. pombe* Δ*coq11* strain produced only ∼2% of the CoQ_10_ level of the WT strain indicated that *S. pombe* Coq11 is also responsible for CoQ_10_ synthesis. We designated *SPAC1071.11* as *coq12* because this gene deletion also lowered the CoQ level to ∼4% of the WT level. Both *coq11* and *coq12* are very important for CoQ_10_ synthesis but are not absolutely required for its synthesis, unlike *dps1*, *dlp1*, and *coq2–coq9* genes. To confirm that the strains obtained from Bioneer Corp do not have any extra mutations that might affect CoQ_10_ production, we created Δ*coq11* and Δ*coq12* strains ourselves and obtained the same results, showing low production of CoQ_10_ in Δ*coq11* and Δ*coq12* strains.

**Table 1:** Newly discovered genes responsible for significant CoQ loss

Gene name	Function of the gene products	CoQ10 (μg/1 × 109 cells) (disruptant/WT)
*coq11* (*SPCC1840.09*)	NAD-dependent epimerase/dehydratase family protein (ubiquinone biosynthesis protein Coq11)	0.015 ± 0.01
*coq12* (*SPAC1071.11*)	NADH-dependent flavin oxidoreductase (predicted)	0.038 ± 0.01
*ppr6*	Mitochondrial PPR repeat protein Ppr6	0.188 ± 0.02
*por1*	Mitochondrial outer membrane voltage-dependent anion-selective channel Por1	0.189 ± 0.03
*ppm1*	Leucine carboxyl methyltransferase, involved in regulation of PPA2	0.196 ± 0.03
*atp12*	Mitochondrial F_1_–F_0_ ATP synthase chaperone Atp12	0.257 ± 0.07
*qcr9*	Ubiquinol-cytochrome *c* reductase complex subunit 9	0.288 ± 0.02
*cbp6*	Mitochondrial complex III assembly protein Cbp6	0.319 ± 0.03
*cox5*	Cytochrome *c* oxidase subunit V	0.337 ± 0.02
*mpa1*	Mitochondrial translational activator Mpa1	0.362 ± 0.08

Δ*ppr6* and Δ*atp12* were performed with n = 3; Δ*coq11*, Δ*coq12*, Δ*por1*, Δ*ppm1*, Δ*qcr9*, Δ*cbp6*, Δ*cox5*, and Δ*mpa1* were performed with n = 2.

### Phenotypes of Δ*coq11* and Δ*coq12* strains

**Figure 2:**
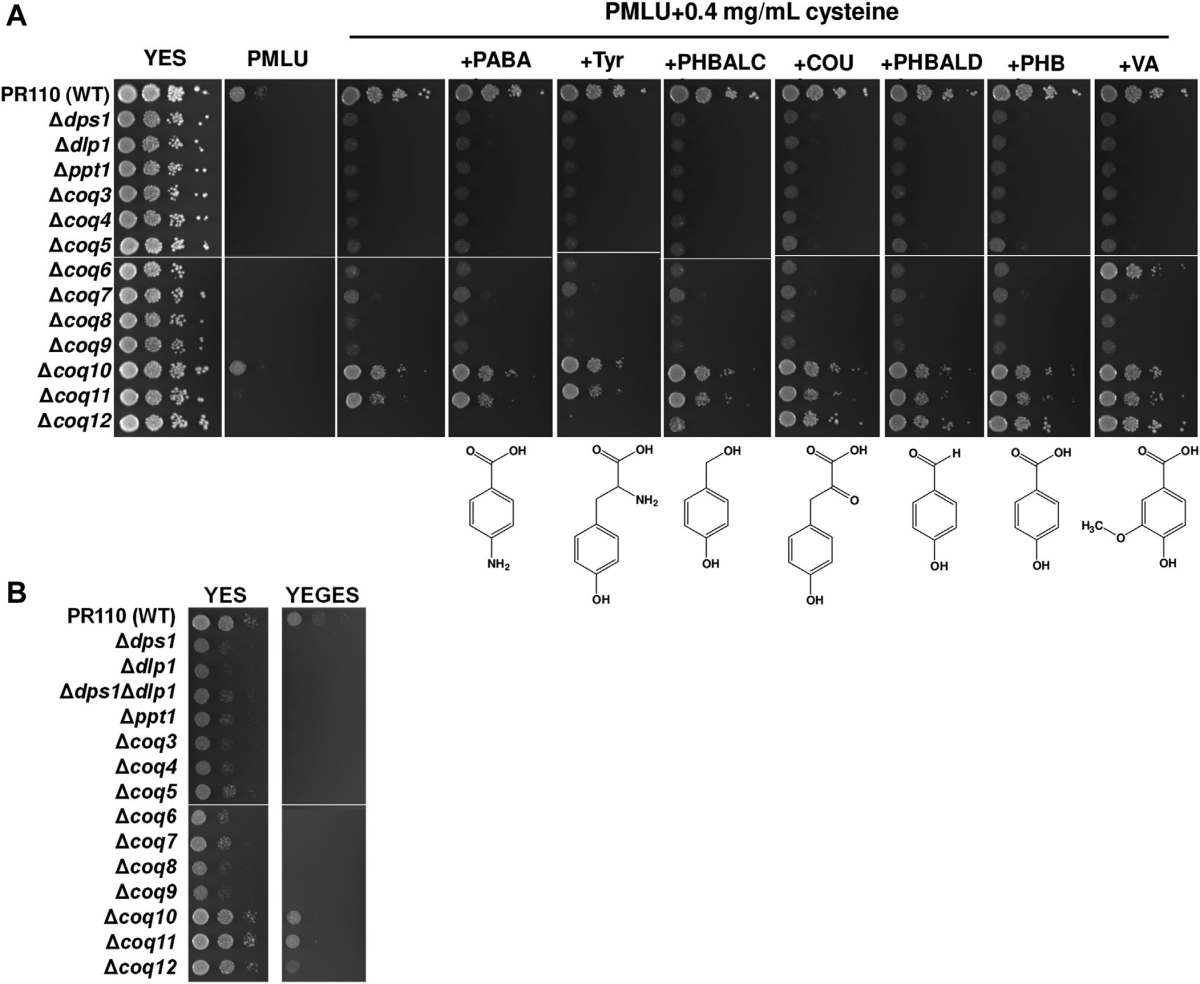
**Growth phenotype of the *Schizosaccharomyces pombe* Δ*coq11*, Δ*coq12*, and other *coq* disruptants.***A*, effect of putative precursors upstream of quinone synthesis on the growth of Δ*coq11*, Δ*coq12*, and other *coq* disruptants was tested. Growth of the *S. pombe* WT strain PR110 and Δ*dps1*, Δ*dlp1*, Δ*ppt1*, Δ*coq3*, *4*, *5*, *6*, *7*, *8*, *9*, *10*, *11*, *12* with YES or PMLU base minimal media (225 mg/ml of leucine and uracil were added to the minimal media). About 100 μg/ml of each aromatic chemical was added if necessary). About 2.0 × 10^6^ cells/ml of each strain and serial dilution of 10^−1^ to 10^−4^ (from *left* to *right*) were spotted onto the agar media and grew 4 days. *B*, growth phenotype of the *coq* disruptants on nonfermentable carbon source was also tested. The indicated strains were spotted onto YES (3% glucose) and YEGES (2% glycerol + 1% ethanol [w/v]). About 1 day-grown preculture was used for the assay. Absorbance of 2 at 600 nm of each strain and serial dilution of 10^−1^ to 10^−2^ (from *left* to *right*) were spotted onto the agar plates, and the photos were taken at 2 days (YES) or 8 days (YEGES). A similar trend was observed in the other dataset including previous study (10). COU, *p*-coumarate; cys, cysteine; PABA, *p*-aminobenzoic acid; PHB, *p*-hydroxybenzoic acid; PHBALC, *p*-hydroxybenzyl alcohol (gastrodigenin); PHBALD, *p*-hydroxybenzaldehyde; Tyr, tyrosine; VA, vanillic acid; YES, yeast extract with supplement.

**Figure S1:**
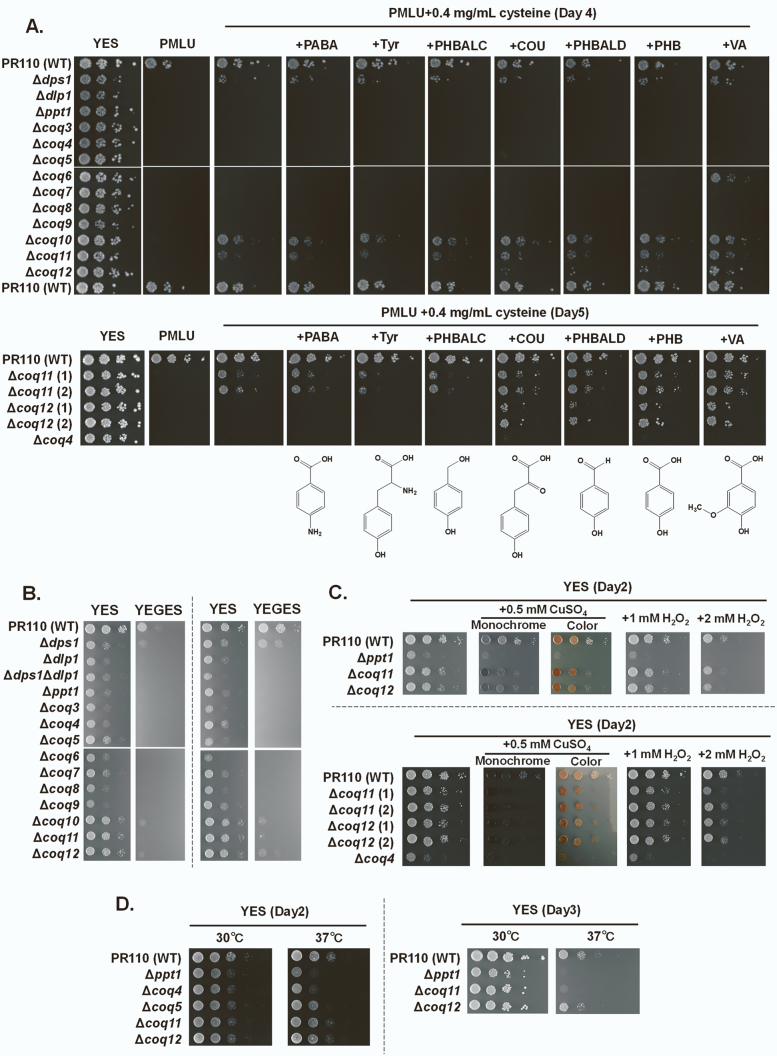
**Reproducibility of**Figure 2 and 3 **experiments.***A*, the experiment was similarly performed as Figure 2*A*. Growth of the *S. pombe* Growth of the *S. pombe* wild-type strain PR110 and Δ*dps1*, Δ*dlp1*, Δ*ppt1*, Δ*coq3*, *4*, *5*, *6*, *7*, *8*, *9*, *10*, *11*, *12*: *upper panel*; or PR110, Δ*coq11*, *12*, and *4* (for Δ*coq11* and *12*, two independent single colonies were used): *lower panel* with YES or PMLU base minimal media (PMLU_75_: 75 mg/ml of leucine and uracil were added to the minimal media). 100 μg/ml of each aromatic chemical was added if necessary. 2.0 × 10^6^ cells/ml of each strain and serial dilution of 10^−1^ to 10^−3^ (from *left* to *right*) were spotted onto the agar media and grew 4 or 5 days. *B*, the experiments were similarly performed as Figure 2*B*. The strains used in Figure 2*B* were spotted onto YES (3% glucose) or YEGES (2% glycerol + 1% ethanol (w/v)). OD_600_ = 2 of each strain and serial dilution of 10^−1^ to 10^−2^ (from *left* to *right*) were spotted onto the agar plates and the photos were taken at 2 (YES) or 8 days (YEGES). *C*, the experiments were similarly performed as Figure 3 or *A*. Strains were spotted onto YES or chemical additive media (CuSO_4_: 0.5 mM, H_2_O_2_: 1 mM or 2 mM). OD_600_ = 2 of each strain and serial dilution of 10^−1^ to 10^−3^ (from *left* to *right*) were spotted onto the agar plates and the photos were taken at 2 days. For the CuSO_4_ plate, monochrome and color images are taken. *D*, the experiment was similarly performed as Figure 3. Growth of the *S. pombe* wild-type strain PR110, Δ*ppt1*, Δ*coq4*, Δ*coq5*, Δ*coq11*, and Δ*coq12* on YES (*left panel*) or PR110, Δ*ppt1*, Δ*coq11*, and Δ*coq12* on YES (*right panel*). OD_600_ = 2 of each strain and serial dilution of 10^−1^ to 10^−4^ (from *left* to *right*) were spotted onto the agar plates and grew for two days at 30 °C and ∼37 °C (*left panel*) or 3 days at 30 °C and 37 °C (*right panel*).

We first noticed that Δ*coq11* and Δ*coq12* strains did not grow well on minimal medium, as was observed for CoQ-deficient *S. pombe* (Figs. 2*A* and S1*A*). We then tested the effect of PHB and related compounds on the growth of deletion mutants to see how these products affect their growth. Growth phenotypes for each of the single disruptants of all CoQ biosynthetic genes and the *coq10* gene encoding CoQ-binding protein were assessed by spot tests on yeast extract with supplement (YES) complete medium and Pombe minimal with leucine and uracil (PMLU) medium with indicated additives (Figs. 2*A* and S1*A*). All strains including WT grew well on YES complete medium during 4 days of incubation. On PMLU minimal medium, all tested strains except WT (and the Δ*coq10* strain) did not grow well. Like other mutants lacking CoQ, the Δ*coq11* strain showed better growth on cysteine-containing medium. By contrast, the Δ*coq12* strain showed almost no growth on PMLU medium containing cysteine. The requirement of cysteine for growth in CoQ-less mutants has been observed previously (10), but it was different between c*oq11* and *coq12* disruptants. Addition of PHB, its aldehyde form PHBALD, *p*-coumarate, and vanillic acid (VA) restored the slow growth phenotype of the Δ*coq12* strain, whereas no clear effect of these compounds was observed on the growth of the Δ*coq11* strain. Addition of the alcoholic form *p*-hydroxybenzyl alcohol (PHBALC), PABA, or tyrosine had almost no growth recovery effect on the Δ*coq12* strain. Based on these results, we predicted that Coq11 and Coq12 are involved in different steps in the CoQ synthetic pathway. We found that addition of VA restored growth of the *S. pombe* Δ*coq6* strain, as was similarly observed for the *S. cerevisiae coq6* disruptant (30) (Figs. 2*A* and S1*A*).

We next tested the growth of the mutants on medium containing the nonfermentable carbon source YEGES, which contains yeast extract, ethanol, and glycerol. The Δ*dps1*, Δ*dlp1*, Δ*ppt1*, Δ*coq3*, Δ*coq4*, Δ*coq5*, Δ*coq6*, Δ*coq7*, Δ*coq8*, and Δ*coq9* single mutants and the Δ*dps1*Δ*dlp1* double mutant were unable to grow on YEGES medium (Figs. 2*B* and S1*B*) as previously described (10); this phenotype indicates that these strains are respiration defective. The Δ*coq10*, Δ*coq11*, and Δ*coq12* strains grew (Figs. 2*B* and S1*B*) but slower than the WT strain on the same medium, indicating that these strains are partially respiration defective.

**Figure 3:**
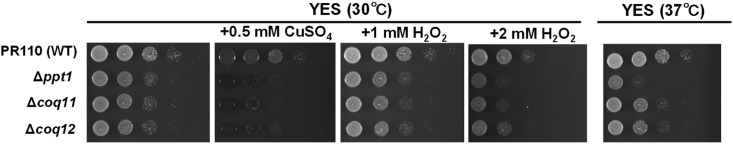
**Effect of various stresses in *Schizosaccharomyces pombe* WT, Δ*ppt1*, Δ*coq11*, and Δ*coq12*.***S. pombe* strains were spot onto YES or exhibited chemical additive media (CuSO_4_: 0.5 mM, H_2_O_2_: 1 mM or 2 mM). More than 1 day-grown preculture was used for the assay. Absorbance of 2 at 600 nm of each strain and serial dilution of 10^−1^ to 10^−4^ (from *left* to *right*) was spotted onto the agar media and grew 2 days at 30 °C or 37 °C. YES, yeast extract with supplement.

**Figure 4:**
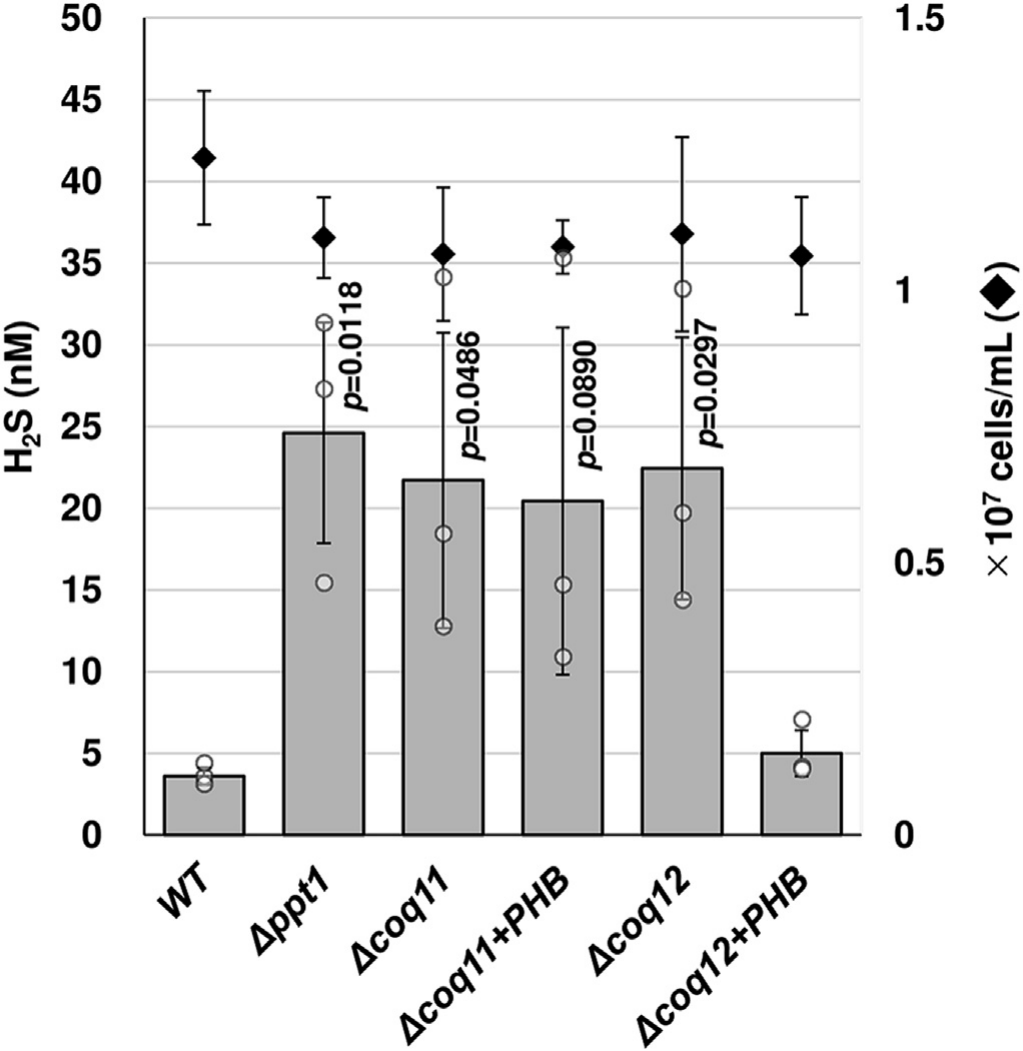
**Comparison of hydrogen sulfide (H**_**2**_**S) concentration under various condition.***Schizosaccharomyces pombe* cells were grown in YES (WT PR110, Δ*coq11*, and Δ*coq12*) or YES with 100 μg/ml PHB (Δ*coq11* and Δ*coq12*) for 28 h (late log phase), and H_2_S concentrations were measured by the method described previously (31). Data are represented as the mean ± SD of three measurements. The corresponding actual values are shown as plots. The exact *p* values on the bars denote statistically significant differences or approaching significance relative to samples from WT (Student’s *t* test). *Diamonds* show cell number (*right vertical axis*). PHB, *p*-hydroxybenzoic acid; YES, yeast extract with supplement.

Our previous results showed that CoQ-less mutants displayed differences in stress sensitivity to hydrogen peroxide (H_2_O_2_) and CuSO_4_ (10). Therefore, we next investigated the sensitivity to H_2_O_2_ and CuSO_4_ in the Δ*coq11* and Δ*coq12* strains. Similar to the Δ*coq2* (*ppt1*) strain, the Δ*coq11* and Δ*coq12* strains grew more slowly in the presence of 1 and 2 mM H_2_O_2_ or 0.5 mM CuSO_4_ than in its absence (Figs. 3 and S1*C*), whereas the WT strain was not affected at the concentrations tested. By contrast, Δ*coq11* and Δ*coq12* strains grew better than the Δ*coq2* (*ppt1*), Δ*coq4*, and Δ*coq5* strains at high temperature (37 °C: Figs. 3 and S1*D*). These results suggest that the CoQ level of the Δ*coq11* and Δ*coq12* strains is not enough to tolerate oxidative or copper ion stress, but it is sufficient for growth on media containing nonfermentable carbon sources or at higher temperatures. In the same experiment, we noticed dark color pigment of CuS in the Δ*coq11* and Δ*coq12* strains (Figs. 3 and S1*C*), which has been observed in other *coq* gene disruptants (10). This is consistent with the results showing that CoQ-deficient *S. pombe* strains produced more sulfide (hydrogen sulfide) because of loss of sulfide–quinone oxidoreductase functionality (31). To confirm this, we measured sulfide levels in Δ*coq11* and Δ*coq12* strains (Fig. 4). The results revealed higher sulfide levels in both Δ*coq11* and Δ*coq12* strains, and addition of PHB to the Δ*coq12* strain, but not the Δ*coq11* strain, significantly decreased sulfide production (Fig. 4).

### Coq12 is involved in the PHB biosynthetic pathway

**Figure 5:**
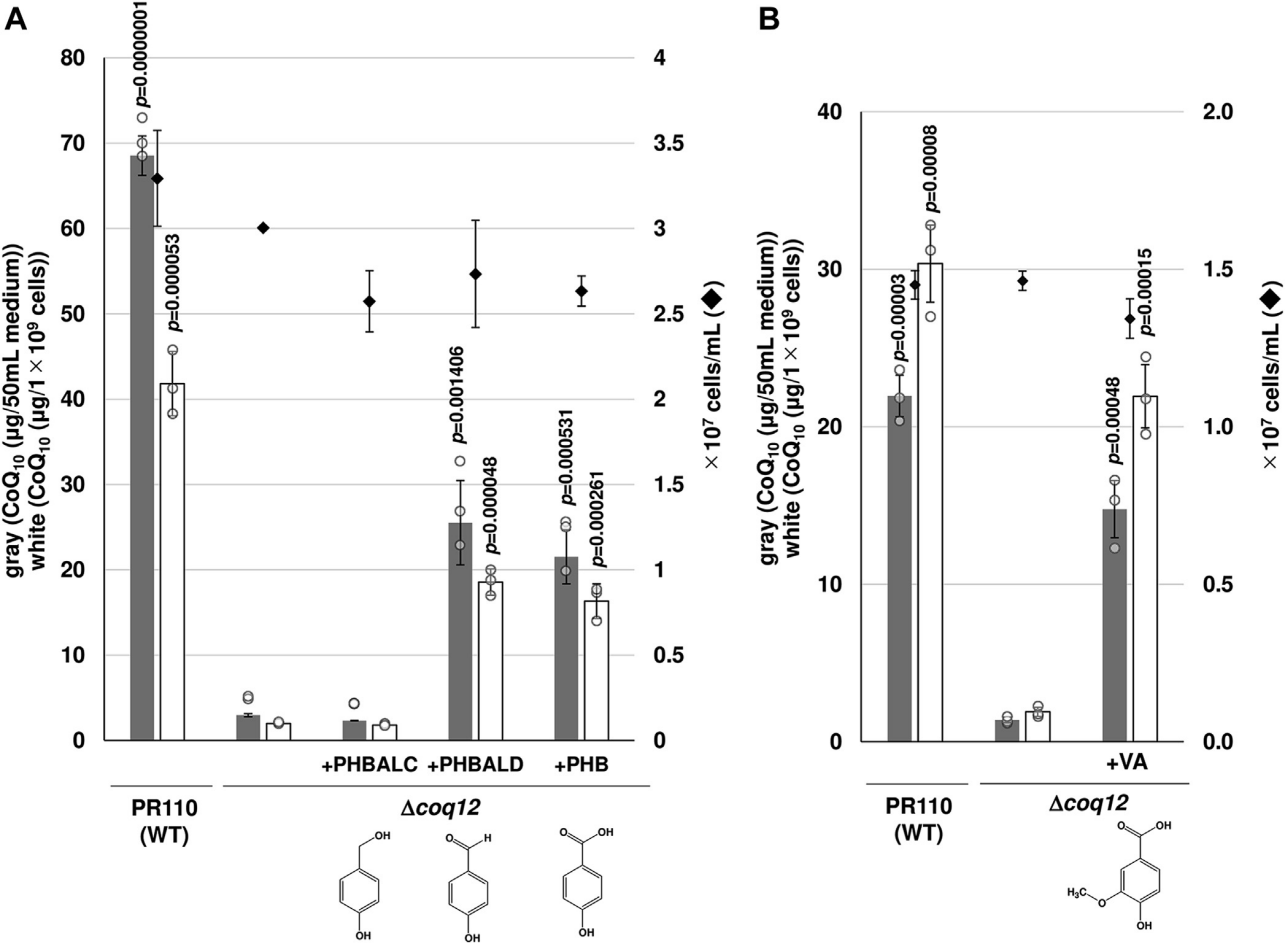
**Effect of PHB, PHBALC, PHBALD, and VA on CoQ**_**10**_**production of Δ*coq12*.** WT (PR110) and Δ*coq12* cells were cultivated for 48 h at 30 °C. The Δ*coq12* cells were also cultivated in YES with 100 μg/ml PHBALC, PHBALD, PHB (*A*), or VA (*B*). *Gray bars* show the CoQ_10_ content per 50 ml of medium, and *white bars* show CoQ_10_ normalized by cell number (*left vertical axis*). *Diamonds* show cell number (*right vertical axis*). Five micrograms of CoQ_6_ was used as an internal standard. Data are represented as the mean ± SD of three measurements. The corresponding actual values are shown as plots. The exact *p* values on the bars denote statistically significant increase relative to samples from Δ*coq12* (Student’s *t* test). PHB, *p*-hydroxybenzoic acid; PHBALC, *p*-hydroxybenzyl alcohol; PHBALD, *p*-hydroxybenzaldehyde; VA, vanillic acid; YES, yeast extract with supplement.

As shown previously, PHB, PHBALD, and VA restored the slow growth of the Δ*coq12* strain on minimal medium (Fig. 2*A*). We next tested whether these compounds affect the production of CoQ_10_ (Fig. 5*A*). The results showed that addition of 100 μg/ml of PHB or PHBALD, an aldehyde form, restored CoQ_10_ production in the Δ*coq12* strain, but PHBALC, an alcoholic form, did not. VA addition also restored CoQ_10_ production in the Δ*coq12* strain (Fig. 5*B*). These results suggest that Coq12 is involved in some upstream reaction to produce PHBALD or PHB.

**Figure S2:**
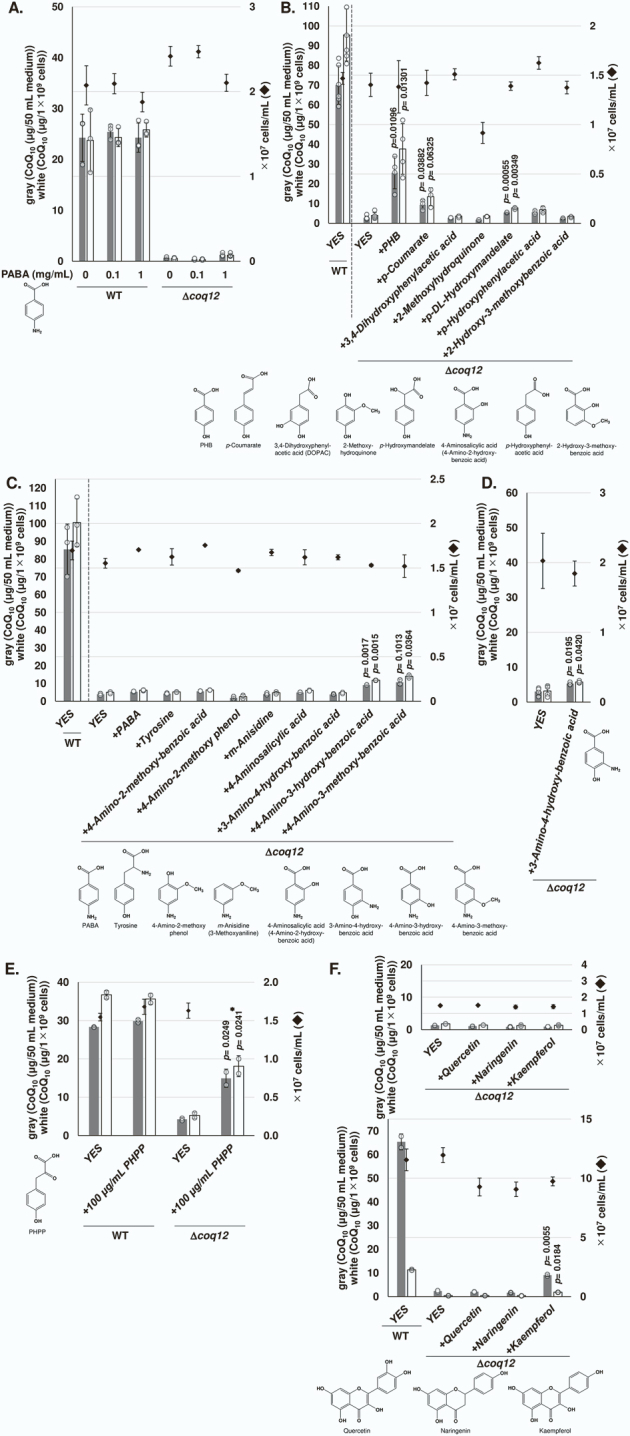
**Effect of various aromatic compounds on CoQ**_**10**_**production in Δ*coq12*.** WT PR110 and Δ*coq12* (IN1) cells were pre-cultivated in 10 ml YES medium for 1 day. Cells at an initial density of ∼1 × 10^5^ cells/ml were cultivated in the presence of aromatic compounds, with rotation at 30 °C. Gray bars show the CoQ_10_ content per 50 ml of medium, and white bars show CoQ_10_ normalized by cell number. Diamonds show cell number. Five micrograms of CoQ_6_ was used as an internal standard. *A*, indicated amount of PABA was added to the medium of growing the WT or Δ*coq12* cells. Data are represented as the mean ± SD of three measurements. The corresponding actual values are shown as plots. *B*, 1 mg/ml of PHB, *p*-coumarate, 3,4-dihydroxyphenylacetic acid, DL-*p*-hydroxymandelic acid, *p*-hydroxyphenylacetic acid, or 2-hydroxy-3-methoxybenzoic acid, or 0.5 mg/ml of 2-methoxyhydroquinone was added to the medium of growing Δ*coq12* cells. Data are represented as the mean ± SD of six (WT in YES or Δ*coq12* in YES), four (Δ*coq12* in YES +PHB), three (Δ*coq12* in YES +*p*-coumarate), or two (Δ*coq12* in YES +3,4-dihydroxyphenylacetic acid, 2-methoxyhydroquinone, DL-*p*-hydroxymandelic acid, *p*-hydroxyphenylacetic acid, or 2-hydroxy-3-methoxybenzoic acid) measurements. The corresponding actual values are shown as plots. The exact *p* values on the bars denote statistically significant increase relative to samples from the Δ*coq12* grown in YES (Student’s *t*-test). *C*, 200 μg/ml of PABA, L-tyrosine, 4-amino-2-methoxybenzoic acid, 4-amino-2-methoxy phenol, *m*-anisidine hydrochloride, 4-aminosalicylic acid (4-amino-2-hydroxybenzoic acid), 3-amino-4-hydroxybenzoic acid, 4-amino-3-hydroxybenzoic acid, or 4-amino-3-methoxybenzoic acid was added to the medium of growing Δ*coq12* cells. Data are represented as the mean ± SD of three (WT in YES or Δ*coq12* in YES), or two (Δ*coq12* in YES +the amino group containing aromatic compounds) measurements. The corresponding actual values are shown as plots. The exact *p* values on the bars denote statistically significant increase relative to samples from the Δ*coq12* grown in YES (Student’s *t*-test). *D*, 1 mg/ml of 3-amino-4-hydroxybenzoic acid was added to the medium of growing Δ*coq12* cells. Data are represented as the mean ± SD of four (Δ*coq12* in YES), or three (Δ*coq12* in YES +3-amino-4-hydroxybenzoic acid) measurements. The corresponding actual values are shown as plots. The exact *p* values on the bars denote statistically significant increase relative to samples from the Δ*coq12* grown in YES (Student’s *t*-test). *E*, 100 μg/ml of PHPP (*p*-hydroxyphenylpyruvate) was added to the medium of growing the WT or Δ*coq12* cells. Data are represented as the mean ± SD of two measurements. The corresponding actual values are shown as plots. The exact *p* values on the bars denote statistically significant increase relative to samples from the Δ*coq12* grown in YES (Student’s *t*-test). *E*, 100 μg/ml of quercetin, naringenin, or kaempferol was added to the medium of growing the Δ*coq12* cells (*upper graph*: Oxoid Yeast Extract Lot No. 4325105-02, lower graph: Lot No. 2198213–02). Data are represented as the mean ± SD of two measurements. The corresponding actual values are shown as plots. The exact *p* values on the bars denote statistically significant increase relative to samples from the Δ*coq12* grown in YES (Student’s *t*-test).

To identify the point of deficiency of the reaction upstream of PHB synthesis in the Δ*coq12* strain, we tested various compounds that may affect PHB synthesis (Fig. S2). Among the tested compounds, *p*-coumarate, *p*-hydroxyphenylpyruvate, and dl-*p*-hydroxymandelic acid slightly restored CoQ_10_ levels in the Δ*coq12* strain (Fig. S2, *B* and *E*), but CoQ_10_ production was ∼30% of the level when PHB was added. Hydroxy groups or methoxy groups possessing amino benzoic acids such as 3-amino-4-hydroxybenzoic acid, 4-amino-3-hydroxybenzoic acid, or 4-amino-3-methoxybenzoic acid very slightly restored CoQ_10_ production in the Δ*coq12* strain (Fig. S2, *C* and *D*). Addition of PABA, 3,4-dihydroxyphenylacetic acid, 4-aminosalicylic acid, 4-amino-2-methoxybenzoic acid, 4-amino-2-methoxy phenol, *m*-anisidine, or 2-hydroxy-3-methoxybenzoic acid did not restore CoQ_10_ levels in the Δ*coq12* strain (Fig. S2, *A*–*C*). In plants and mammals, kaempferol and other related compounds are known to be involved in the alternative pathway of CoQ biosynthesis (32, 33). Therefore, we tested the effects of flavonoids quercetin, naringenin, and kaempferol on CoQ_10_ production in the Δ*coq12* strain (Fig. S2*F*). The results showed that addition of kaempferol elevated CoQ_10_ production in the Δ*coq12* strain that grew to four times that without (Fig. S2*F*, *lower graph*), whereas quercetin and naringenin had no effect. We also noticed that the effect of kaempferol was not observed when yeast extract of Lot no. 4325105-02 (Fig. S2*F*, *upper graph*) was used instead of Lot no. 2198213-02.

**Figure 6:**
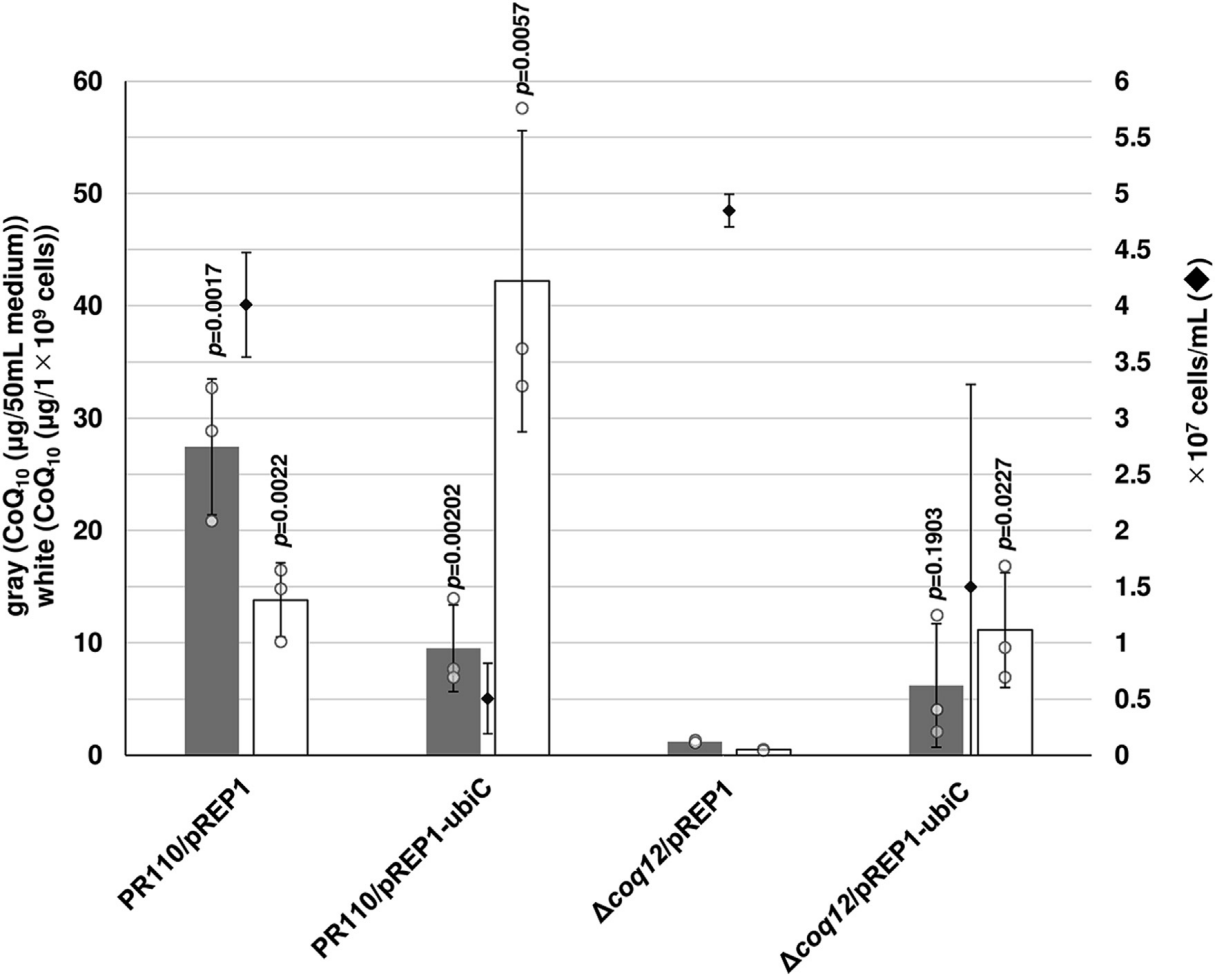
**Overexpression of *ubiC* and its effect on the CoQ**_**10**_**production in Δ*coq12*.** The WT PR110 and Δ*coq12* (IN1) cells harboring pREP1 or pREP1-ubiC were cultivated in PMU medium for 72 h at 30 °C. *Gray bars* show the CoQ_10_ content per 50 ml of medium, and *white bars* show CoQ_10_ normalized by cell number. *Diamonds* show cell number. Five micrograms of CoQ_6_ was used as an internal standard. Data are represented as the mean ± SD of three measurements. The corresponding actual values are shown as plots. The exact *p* values on the bars denote statistically significant increase relative to samples from the Δ*coq12*/pREP1 (Student’s *t* test). PMU, Pombe minimal medium containing uracil but lacking leucine.

*E. coli* UbiC is an enzyme that removes the pyruvyl group from chorismate, with concomitant aromatization of the ring, to provide PHB for CoQ synthesis. Here, we tested heterologous expression of *ubiC* (34) in the Δ*coq12* strain, expecting that the *ubiC* gene might recover CoQ production. Overexpression of *ubiC* caused growth inhibition in WT, lowering the CoQ content per volume in the WT strain (Fig. 6). By contrast, overexpression of *ubiC* in the Δ*coq12* strain restored the CoQ_10_ level per volume and per cell number. This result indicates that UbiC supports PHB production in *S. pombe*, which is also consistent with the fact that supplementation of PHB restored CoQ_10_ levels in the Δ*coq12* strain (Fig. 5).

### Disruption of Coq11 or Coq12 lowers Coq4 protein levels

**Figure 7:**
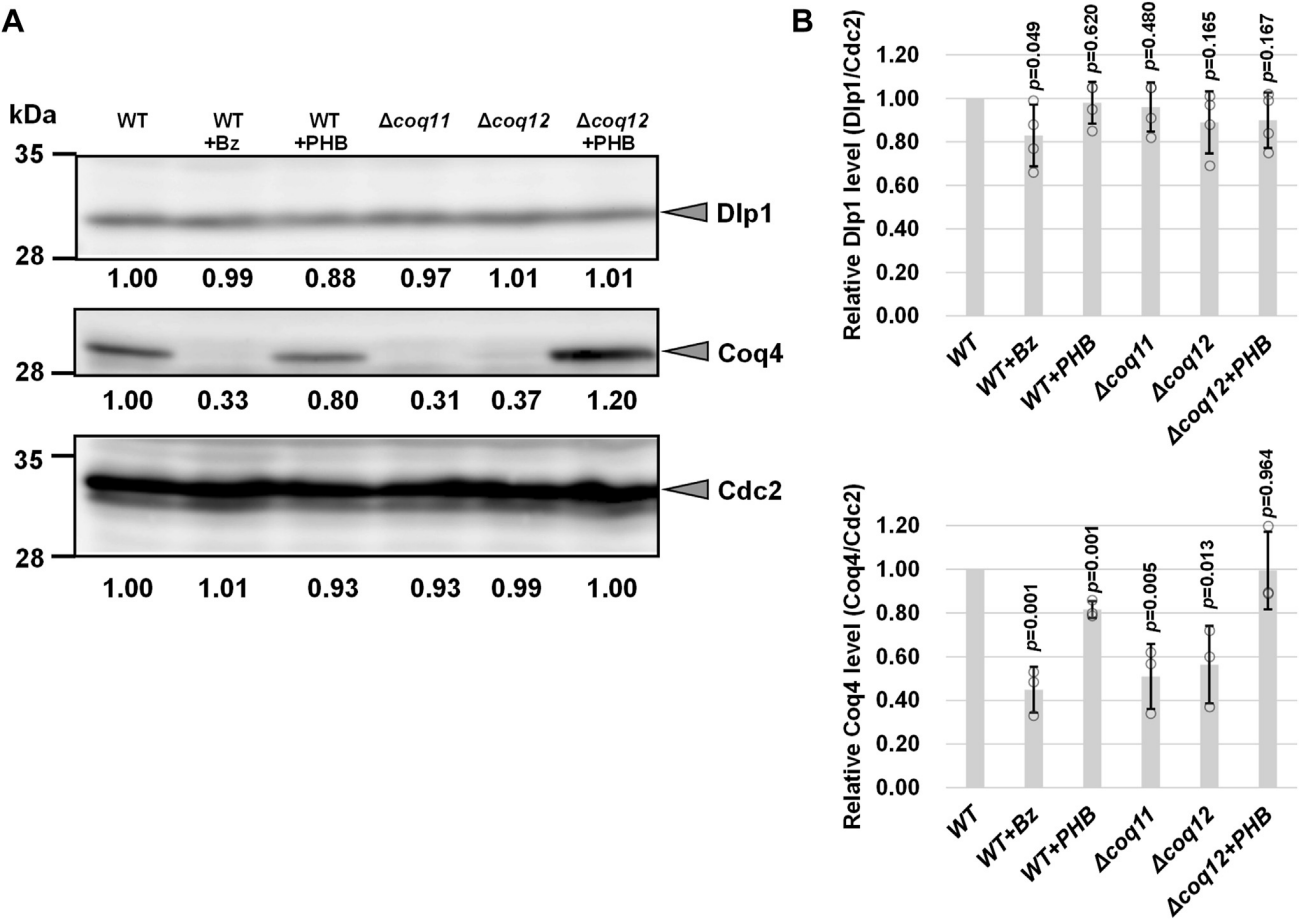
**Expression level of Dlp1 and Coq4 in WT, Δ*coq11*, and Δ*coq12*.***A*, for the preculture, the yeast cells were cultivated in 10 ml YES for 1 day. Yeast cells were cultivated in 55 ml YES at an initial cell density of 1 × 10^6^ cells/ml and cultivated for 24 h with rotation at 30 °C. Protein was extracted as experimental procedure. Each sample was subjected to 10% SDS-polyacrylamide gel electrophoresis and analyzed by immunoblotting using rabbit antibodies against Dlp1, Coq4, and Cdc2. Western blotting of Coq4 and Dlp1 in WT, Δ*coq11*, and Δ*coq12*. Dlp1, Coq4, and Cdc2 as a loading control for whole cells were analyzed by Western blotting. Target proteins are indicated on the *right*. The yeast strain and chemical additives in each lane are shown at the *top*. Lane 1, PR110 (WT); lane 2, PR110 (WT) +100 μg/ml benzoic acid; lane 3, WT +100 μg/ml PHB; lane 4, RYP26 (Δ*coq11*); lane 5, IN1 (Δ*coq12*); and lane 6, IN1 (Δ*coq12*) + 100 μg/ml PHB. The amount of proteins was quantified by ImageJ, and relative Dlp1 and Coq4 levels (Dlp1/Cdc2 and Coq4/Cdc2) were calculated. A similar trend was observed in our separate experiments (Fig. S3). *B*, data are represented as the mean ± SD of four (Dlp1/Cdc2) or three (Coq4/Cdc2) measurements. The corresponding actual values are shown as plots. The exact *p* values are shown relative to the blots of PR110 (WT) (Student’s *t* test). PHB, *p*-hydroxybenzoic acid; YES, yeast extract with supplement.

**Figure S3:**
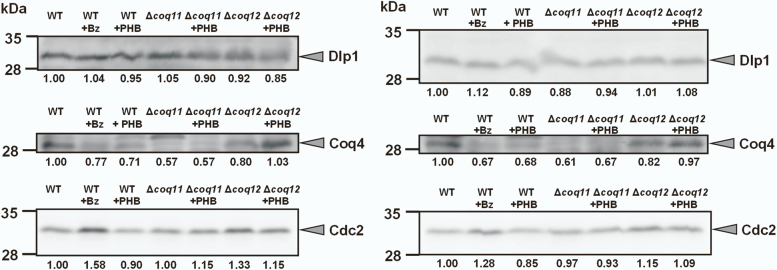
**Reproducibility of**Figure 7**experiments.** The experiments were similarly performed as Figure 7. Western blotting of Coq4 and Dlp1 in WT, Δ*coq11* and Δ*coq12*. Dlp1, Coq4 and Cdc2 as a loading control for whole cells were analyzed by Western blotting. Target proteins are indicated on the *right*. The yeast strain and chemical additives in each lane are shown at the *top*. Lane 1, PR110 (WT); lane 2, PR110 (WT) +100 μg/ml Benzoic acid (Bz); lane 3, WT +100 μg/ml PHB; lane 4, RYP26 (Δ*coq11*); lane 5, RYP26 (Δ*coq11*) +100 μg/ml PHB; lane 6, IN1 (Δ*coq12*); lane 7, IN1 (Δ*coq12*) +100 μg/ml PHB. The amount of proteins were quantified by Image J, and relative Dlp1 and Coq4 levels (Dlp1/Cdc2 and Coq4/Cdc2) were calculated and used for making the graph of Figure 7*B*.

**Figure S4:**
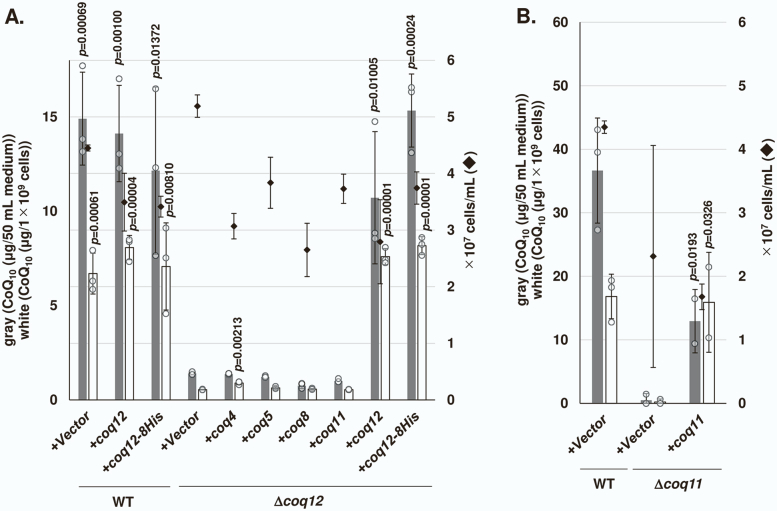
**Overexpression of several *coq* genes and their effect on the CoQ**_**10**_**production in Δ*coq12*.***A*, the WT PR110 cells harboring pREP1 and Δ*coq12* (IN1) cells harboring pREP1, pREP1-coq4, pREP1-coq5, pREP1-coq8, pREP1-coq11, or pREP1-coq12-8xHis were pre-cultivated in 5 ml medium one day (PMU + 0.32 mg/ml cysteine + 0.15 μM thiamine). The yeast cells were cultivated in 55 ml PMU + 0.32 mg/ml cysteine + 0.15 μM thiamine (starting concentration was approx. 1 × 10^5^ cells/ml) and cultivated for 2 days with rotation at 30 °C. Data are represented as the mean ± SD of three measurements. The corresponding actual values are shown as plots. The exact *p* values on the bars denote statistically significant increase relative to samples from the Δ*coq12*/pREP1 (Student’s *t*-test). *B*, the WT PR110 cells harboring pREP1 and Δ*coq11* (RYP26) cells harboring pREP1, or pREP1-coq11 were pre-cultivated in 5 ml medium one day (PMU (WT) or PMU 0.32 mg/ml cysteine +0.15 μM thiamine (Δ*ppt1*)). The yeast cells were cultivated in 55 ml PMU or PMU + 0.32 mg/ml cysteine +0.15 μM thiamine (starting concentration was ∼5 × 10^5^ cells/ml) and cultivated for 2 days with rotation at 30 °C. Data are represented as the mean ± SD of three (WT/pREP1 and Δ*coq11*/ pREP1) or two (Δ*coq11*/ pREP1-coq11) measurements. The corresponding actual values are shown as plots. The exact *p* values on the bars denote statistically significant increase relative to samples from the Δ*coq11*/pREP1 (Student’s *t*-test).

It has been shown that the biosynthetic enzymes responsible for CoQ form a multienzyme complex called the CoQ synthome in *S. cerevisiae* (13), and specific *COQ* gene disruption, especially *COQ4*, lowers the amount of other CoQ proteins in *S. cerevisiae* (35, 36). We previously reported that addition of benzoic acid or deletion of *ppt1* decreased the level of CoQ_10_ in *S. pombe* WT cells and downregulated Coq4 protein production (28). Therefore, the effect of disruption of *coq11* or *coq12* on Coq4 protein levels was analyzed (Figs. 7, *A* and *B* and S3). The amount of Coq4 was significantly reduced in Δ*coq11* and Δ*coq12* single mutants, and also in the WT strain, following benzoic acid treatment (28). Importantly, Coq4 expression was restored in the Δ*coq12* strain cultivated with additional PHB, whereas the Dlp1 protein level, which does not form part of the CoQ protein complex, was not changed in all samples. The effect of overexpression of *coq4*, *coq5*, *coq8*, or *coq11* was also tested in the Δ*coq12* strain, but the CoQ_10_ level was not changed compared with the strain harboring the vector control (Fig. S4*A*). We verified that expression of *coq12* or *coq12-8xHis* in the Δ*coq12* strain and expression of *coq11* in the Δ*coq11* strain fully restored the CoQ_10_ level (Fig. S4, *A* and *B*).

### Coq12 localizes to mitochondria

**Figure 8:**
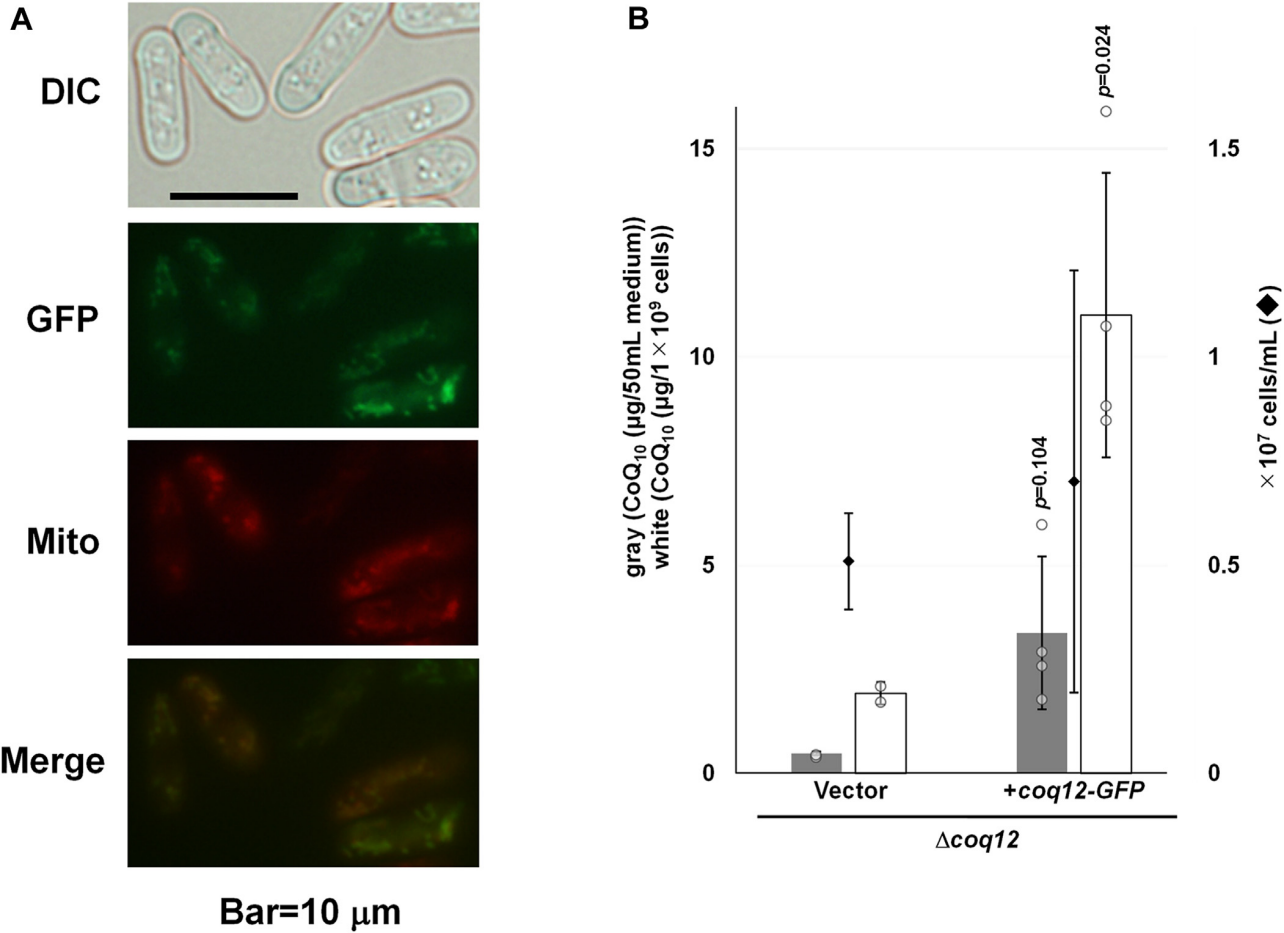
**Subcellular localization of Coq12-GFP by fluorescent microscopy.***A*, differential interference contrast image (DIC), MitoTracker signals (Mito), and GFP signals (Coq12-GFP) are shown. The scale bar represents 10 μm. *B*, complementation assay of *coq12-GFP*. For the preculture, Δ*coq12* (IN1) cells harboring pSLF172LGFPS65A (vector) or pSLF172L-coq12-GFP were cultivated in 10 ml PMU medium 1 day. The yeast cells were cultivated in 55 ml PMU medium (starting concentration was approximately 1 × 10^5^ cells/ml) and cultivated for about 3 days with rotation at 30 °C. Data are represented as the mean ± SD of two (vector) and four (Coq12-GFP) measurements. The corresponding actual values are shown as plots. The exact *p* values on the bars denote statistically significant increase relative to samples from the vector control (Student’s *t* test). PMU, Pombe minimal containing uracil but lacking leucine.

We next investigated the localization of Coq12 to confirm that it is found in mitochondria, like other Coq proteins (10, 14, 15, 35, 36). We constructed a strain chromosomally expressing *coq12-GFP*, but GFP fluorescence was not observed in living cells, although we observed fluorescence when mitochondria were isolated. Therefore, Coq12-GFP was expressed from a plasmid and observed under a fluorescence microscope (Fig. 8*A*). The GFP fluorescence pattern was similar to that of MitoTracker Red, a mitochondria stain. Mitochondrial localization of Coq12 was therefore confirmed, consistent with the results of high-throughput protein localization analysis (37). In addition, CoQ_10_ production was restored in the Δ*coq12* strain expressing Coq12-GFP, confirming that Coq12-GFP is functional (Fig. 8*B*). Thus, Coq12 is involved in CoQ_10_ biosynthesis in mitochondria.

### Domain structure of Coq12

**Figure S5:**
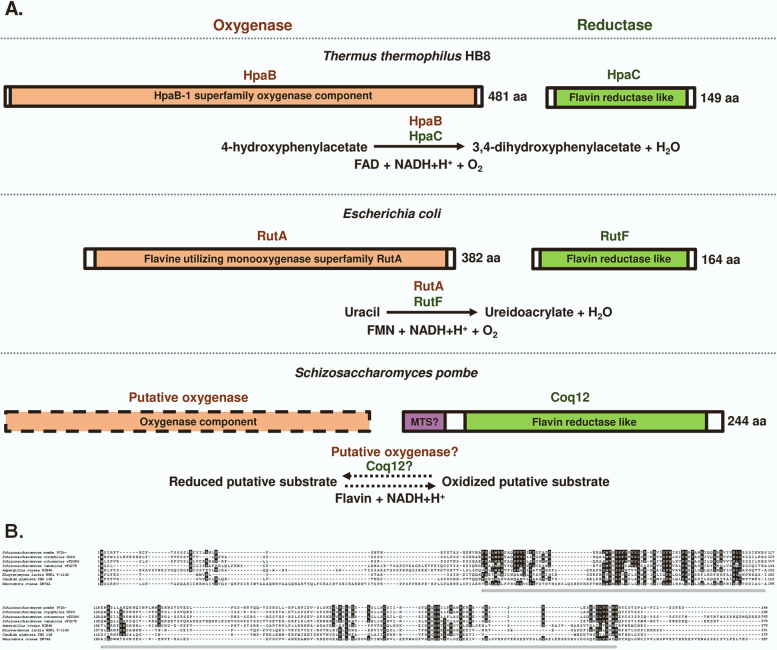
**Putative domain structure of Coq12 and related proteins.***A*, the Coq12 ortholog HpaC reductase, works with HpaB oxygenase to form 3,4-dihydroxyphenylacetate; RutF reductase works with RutA to form ureidoacrylate in their redox reaction. The MTS is predicted from the length of purified Coq12-8xHis and the iPSORT Prediction web site. Coq12 might also work with putative oxygenase(s). The enzymatic function of Coq12 was estimated by the NCBI Conserved Domain Search. The number of amino acid residues for each protein is shown. *B*, alignment of amino acid sequences of *S. pombe* Coq12 (244 amino acid) and the ortholog proteins. The *coq12* orthologs of the *Schizosaccharomyces cryophilus* OY26, *Schizosaccharomyces octosporus* yFS286, *Schizosaccharomyces japonicus* yFS275, *Aspergillus oryzae* RIB40, *Kluyveromyces lactis* NRRL Y-1140, *Candida glabrata* CBS 138, and *Neurospora crassa* OR74A were predicted to encode 235, 260, 279, 279, 221, 215, and 357 amino acid proteins, respectively, and these exhibited 77%, 72%, 58%, 48%, 47%, 44%, and 39% sequence similarity to *S. pombe* Coq12. Amino acid residues that are identical in five or more sequences are indicated by *black boxes*. *Hyphens* indicate the absence of corresponding amino acid residues at those positions. Highly conserved in PNPOx/FlaRed_like superfamily is *gray underlined*. Sequences were aligned using Expresso in T-Coffee Server, which aligns protein sequences using structural information. UniProt IDs are follows: *S. pombe* Coq12, Q9UTQ4; *S. cryophilus*, S9VZB0; *S. octosporus*, S9PXR8; *S. japonicus*, B6JYK7; *A. oryzae*, Q2UUH8; *K. lactis*, Q6CQQ9; *C. glabrata*, Q6FTE2; *N. crassa*, Q1K7L6.

**Figure S6:**
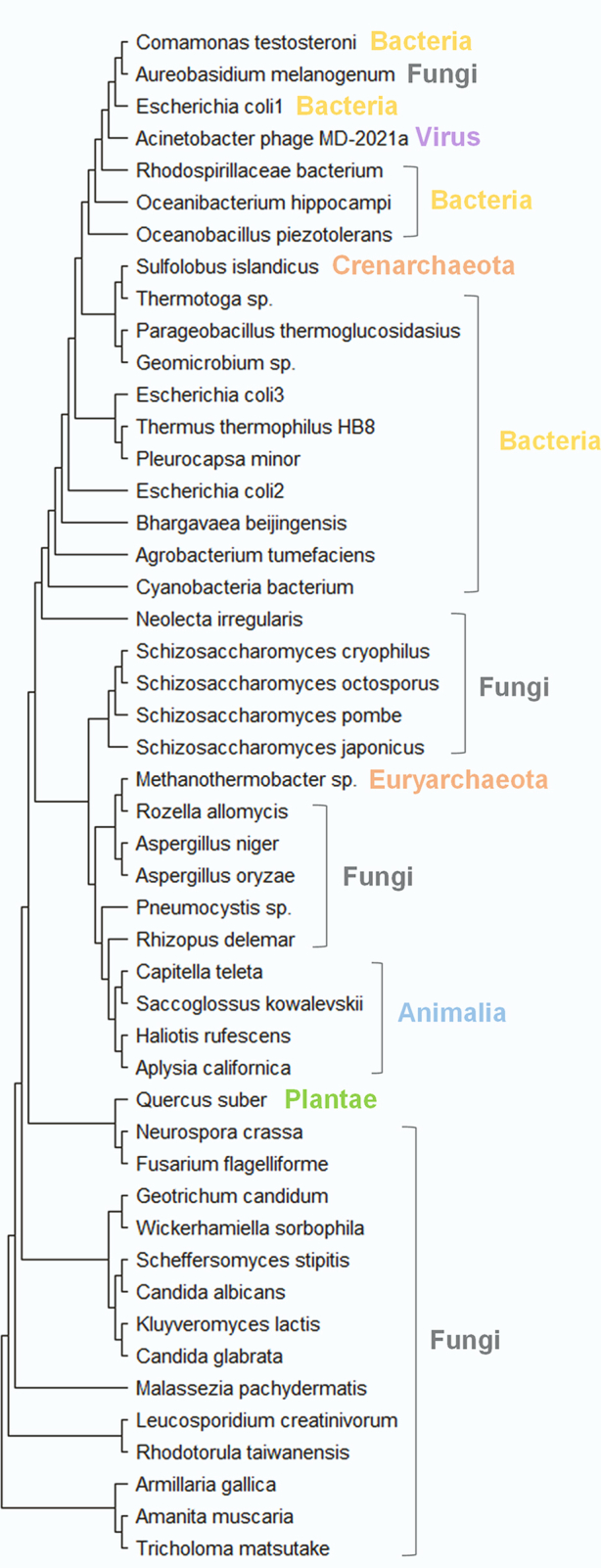
**Phylogeny of Coq12.** Phylogenetic tree of Coq12 and the 47 reductase proteins found in eukaryotes, prokaryotes, or a virus. Homologous proteins of Coq12 were collected *via* NCBI Blast, then the aligned sequence matrix was imported into MEGA11 and analyzed in the Tree Explorer. Classification of kingdom or domain of organisms (or virus) are shown on the *right side*.

A schematic diagram of the primary structure of Coq12 is shown in Fig. S5*A*. Coq12 has a flavin reductase–like domain with a mitochondrial transition signal. The mitochondrial transition signal of Coq12 consists of 30 amino acid residues according to iPSORT Prediction (https://ipsort.hgc.jp/) (38). Based on a conserved domain search using the National Center for Biotechnology Information (39) (https://www.ncbi.nlm.nih.gov/Structure/cdd/wrpsb.cgi), Coq12 (SPAC1071.11) is annotated as a protein categorized to pfam01613 (member of the superfamily cl00381 or PNPOx/FlaRed_like [pyridoxine 5′-phosphate oxidase–like and flavin reductase–like proteins]), a putative mitochondria-localized NADH-dependent flavin oxidoreductase (riboflavin reductase; Enzyme Commission no.: 1.5.1.41 or neighbor enzymes). Most enzymes categorized in pfam01613 are one component of two-component enzymes (40, 41); a smaller component (flavin reductase) and a larger component (a class D flavoprotein monooxygenase) that uses the reduced flavin to hydroxylate substrates (42). The Coq12 ortholog HpaC is a two-component enzyme that works with HpaB to form 3,4-dihydroxyphenylacetate, and similarly, RutF works with RutA to form ureidoacrylate in their redox reaction (Fig. S5*A*). When we searched for orthologs of *S. pombe* Coq12, apparent Coq12 protein orthologs were found to be limited to some fungi (Fig. S5*B*), archaea, bacteria, Annelida, Hemichordata, Mollusca, and viruses (Fig. S6) but absent from some other fungi including *Saccharomyces* yeasts, most green plants, mammals, Euglenozoa, Glomeromycota, Metamonada, Methanococci, and Microspora. It is worth noting that *Kluyveromyces*, *Zygosaccharomyces*, and *Candida glabrata* contain orthologs of Coq12, but their close relative *S. cerevisiae* does not. Moreover, some of Coq12 orthologs have other domains in a single polypeptide such as iron–sulfur cluster binding, DNA or RNA binding, hydroxylase (oxygenase), carbon or arsenite methyltransferase, prenyl transferase, aldehyde dehydrogenase, ferredoxin, and thioredoxin. The limited distribution of Coq12 orthologs and a variety of extra domains in other species suggests that the upstream pathway of CoQ biosynthesis is divergent among species.

### Enzymatic activity of Coq12

**Figure 9:**
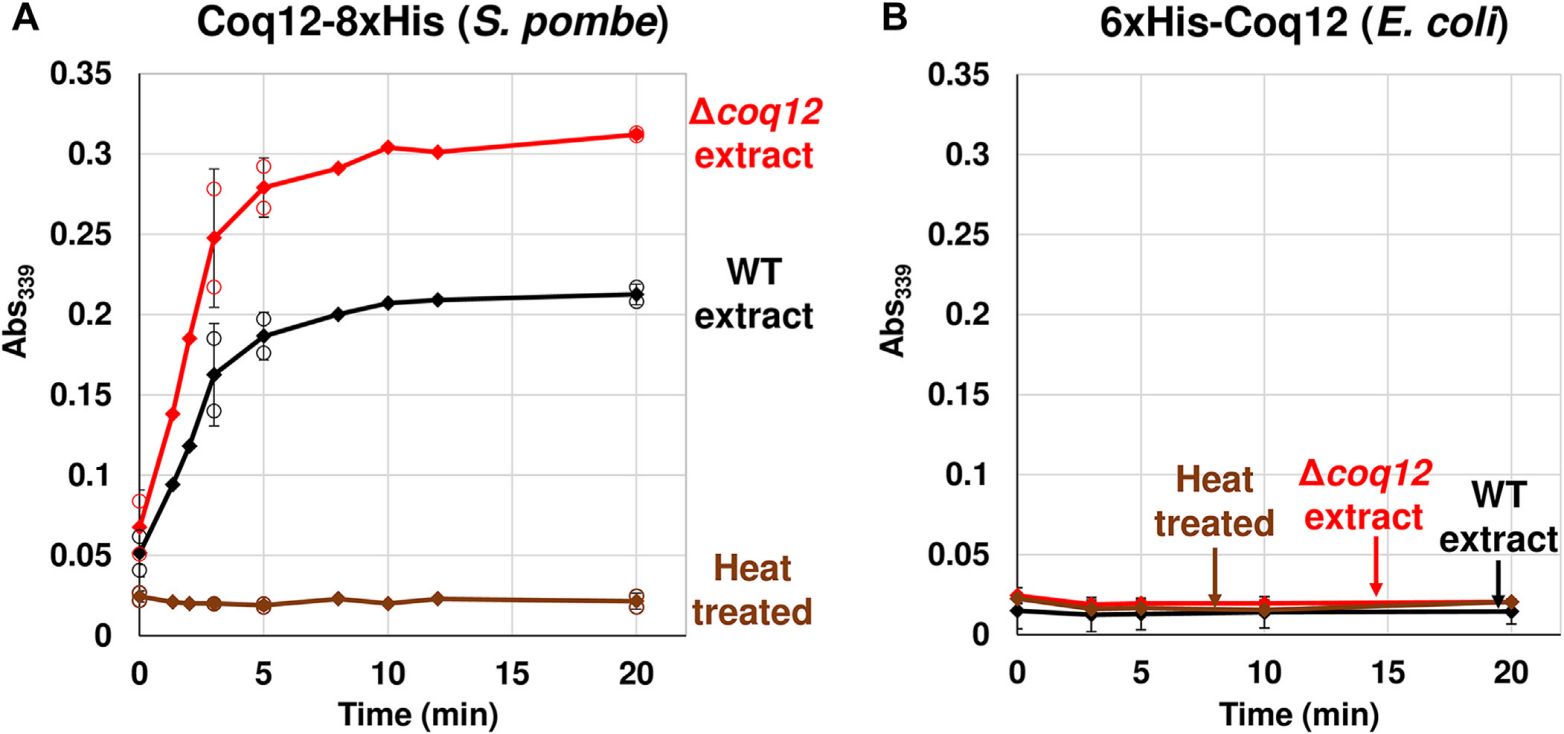
**Enzymatic activity of purified Coq12.** Coq12-8xHis (expressed in *Schizosaccharomyces pombe* and purified (*A*)) or 6xHis-Coq12 (expressed in *Escherichia coli* and purified (*B*)). Yeast cell extract from WT or Δ*coq12* reacted with NAD^+^ and NADH formation was evaluated. Activities on various substrates were determined as described in the Experimental procedures section. The absorbance was measured at 339 nm at the indicated time course. *A*, the absorbance at 0, 3, 5, and 20 min shows the average of two sets of data. *B*, the absorbance at 0, 3, 5, 10, and 20 min shows the average of two sets of data.

**Figure S7:**
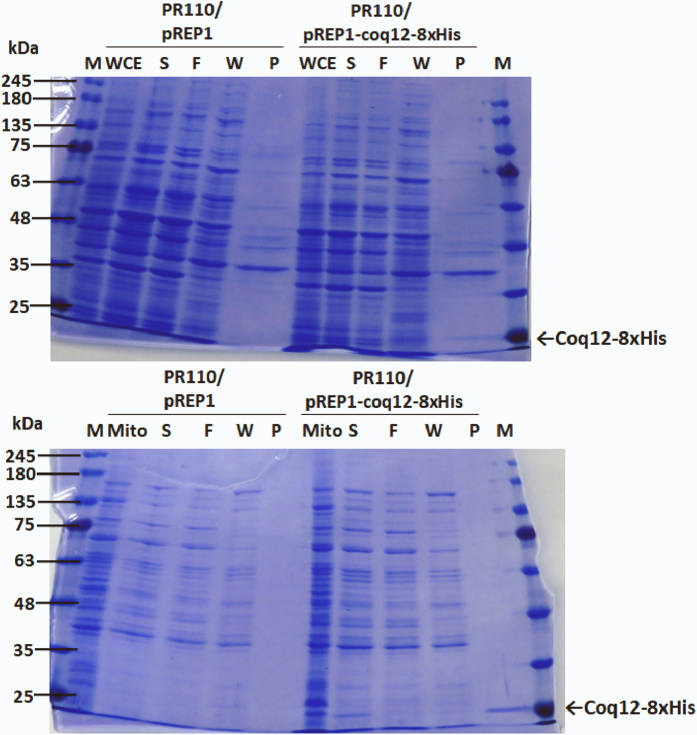
**Confirmation of the purified Coq12-8xHis.** Each sample was subjected to 10.5% SDS–polyacrylamide gel electrophoresis and analyzed by CBB staining. *A*, whole-cell protein profiles were confirmed by CBB staining. The protein was extracted from vector control PR110/pREP1 or Coq12-8xHis expressing cell PR110/pREP1-coq12-8xHis and purified with Ni-NTA Agarose. Fractions: WCE (whole-cell extract), S (supernatant) (cell-free extract), F (flow through), W (wash fraction), P (purified). *Arrow* indicates the estimated Coq12-8xHis band. M: size marker. *B*, isolated mitochondria protein profiles were confirmed by CBB staining. The protein was extracted from vector control PR110/pREP1 or Coq12-8xHis expressing cell PR110/pREP1-coq12-8xHis and purified with Ni-NTA Agarose. Fractions: Mito (isolated crude mitochondria), S (supernatant) (water soluble fraction of mitochondria), F (flow through), W (wash fraction), P (purified). *Arrow* indicates the estimated Coq12-8xHis band. M: size marker

The primary structure of Coq12 encodes predicted NADH oxidation and reduction activity. We therefore expressed and purified His-tagged Coq12 from *S. pombe* and *E. coli* to assess the biochemical properties of Coq12 in NAD^+^ reduction (Fig. S7). When we tested NADH generation from NAD^+^ by purified Coq12 protein from *S. pombe*, a very low level of activity was detected. We assumed that an unknown substrate may need to be included in the reaction. We then extracted ethanol-soluble material from *S. pombe* WT and the Δ*coq12* strains, and crude solutions were mixed with reaction buffer containing NAD^+^ and purified Coq12 to measure the activity. Interestingly, NAD^+^ reduction activity was clearly detected in purified Coq12-8xHis from *S. pombe* (Fig. 9*A*). In particular, incubation of the extract obtained from the Δ*coq12* strain showed higher NAD^+^-reducing activity than the one from the WT strain, suggesting that the Δ*coq12* strain contains a higher amount of an unknown substrate(s) of Coq12. There was no clear NADH production in the reaction with purified Coq12-8xHis incubated with PHBALC, indicating that PHBALC is not a substrate (data not shown). NADH production was absent in the reaction of *S. pombe* extract with Coq12 inactivated by boiling at 95 °C for 1 h. In addition, the Coq12 protein with a 6xHis-tag at the N terminus purified from *E. coli* did not display NADH production when it was reacted with the same *S. pombe* cell extracts (Fig. 9*B*). These results suggest that a partner protein(s) copurified with Coq12 may be required for full NAD^+^ reduction activity.

### Coq12-interacting proteins

**Figure S8:**
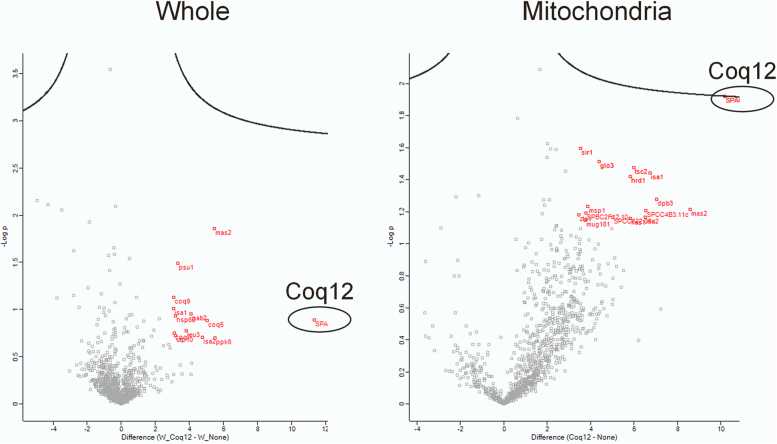
**Candidate of Coq12 interacting proteins.** These volcano plot illustrates significantly differentially abundant proteins. “Whole None” and “Whole Coq12-His” or “Mitochondria None” and “Mitochondria Coq12-His” were compared and abundant protein in Coq12-His group were selected. Coq12 interacting candidate proteins are shown *upper right* of each figure and exhibited by *red font*.

To identify partner proteins of Coq12, proteomic analysis of mitochondrial proteins interacting with purified Coq12-8xHis was performed. His-tagged Coq12 was purified from whole-cell extracts or mitochondria from *S. pombe* and subjected to LC–MS/MS analysis. Using the method described in the Experimental procedures section, 24 proteins were found to interact strongly with Coq12-8xHis (Table S3). Isa1 and Isa2 copurified with Coq12-8xHis (Fig. S8), involved in the formation of iron–sulfur clusters [4Fe–4S] in mitochondria. Mitochondrial processing of peptidase alfa subunit Mas2 was copurified with Coq12-8xHis (Fig. S6). From label-free quantitation intensity analysis, Coq3, Coq4, Coq5, Coq6, Coq7, Coq8, Coq9, and Coq11 were copurified with Coq12x8His purified from mitochondria. Among them, Coq5 was most frequently detected (Tables S4 and S5). In Coq12x8His copurified samples from whole-cell extracts, Coq5, Coq6, Coq7, and Coq9 were detected in LC–MS/MS analysis. Furthermore, Atd1, which is thought to oxidize PHBALD to PHB, was also identified as a binding protein of Coq12x8His purified from mitochondria (Table S5).

## Discussion

The CoQ biosynthetic pathway in eukaryotes is still not fully understood. To better understand CoQ biosynthesis, we searched for fission yeast *S. pombe* gene disruptants containing less CoQ. Among 400 individual gene disruptants, there were no completely null CoQ mutants other than those already known, indicating that there is probably no novel mitochondrial protein strictly required for CoQ synthesis in *S. pombe*, and alternative genes essential for growth may involve CoQ synthesis. Among various gene deletion mutants that produced low levels of CoQ, we studied *coq11* and the newly identified *coq12* mutant because they were the two lowest CoQ_10_ producers (∼2% and ∼4% of WT, respectively). The *COQ11* gene has only been analyzed in *S. cerevisiae*, and herein, we showed that *S. pombe coq11* (34% amino acid similarity with *S. cerevisiae* Coq11p) is important for CoQ synthesis. Instability of Coq4 protein in the *S. pombe* Δ*coq11* strain suggested that Coq11 is closely associated with the CoQ biosynthetic complex. The observation that the Δ*coq11* strain grew better than the other CoQ-less mutants on minimal medium containing cysteine implied that the Δ*coq11* strain still produced CoQ_10_, albeit at a lower level. In the Δ*coq12* strain cultivated with PHB, Coq4 expression was restored, possibly because CoQ_10_ or some intermediate products generated from PHB stabilizes the CoQ biosynthetic complex (synthome).

Phenotypic differences between the Δ*coq11* and Δ*coq12* strains in terms of growth of plates (Figs. 2 and S1*A*) suggest that *coq11* and *coq12* are independently involved in CoQ biosynthesis. Coq12 is a completely uncharacterized protein except for observation of drug sensitivity in large-scale analysis of haploid deletion mutants. In previous reports, *coq12* disruption conferred sensitivity to clotrimazole, terbinafine, and amphotericin B that target ergosterol biosynthesis (43), cadmium sulfate (44), and sodium arsenite (45). We believe that these phenotypes are caused by lowered CoQ_10_ levels in Δ*coq12* cells because the Δ*coq12* strain showed sensitivity to oxidative stress associated with H_2_O_2_ (Figs. 3 and S1*C*).

Several lines of evidence in this study suggest that Coq12 functions upstream of PHB synthesis: (1) addition of PHB or PHBALD restored the growth-defective phenotype and CoQ_10_ synthesis ability of the Δ*coq12* mutant to about half that of WT levels; (2) expression of *E. coli* UbiC, which converts chorismate to PHB, restored CoQ levels in the Δ*coq12* mutant; (3) the Coq12 protein copurified with other CoQ proteins along with putative enzyme Atd1 for converting PHBALD to PHB; and (4) PABA had no effect on restoring CoQ synthesis in the Δ*coq12* mutant. Because fission yeast cells utilize PABA as a substrate for CoQ synthesis (28), lack of PHB synthesis does not completely block CoQ synthesis, indirectly supporting the idea that Coq12 functions upstream of PHB synthesis.

**Figure S9:**
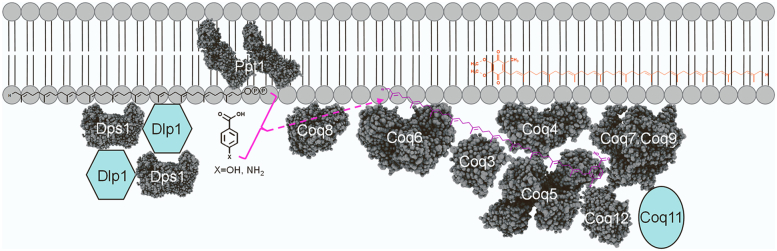
**Structure of the putative CoQ biosynthetic enzyme complex and the estimate**d **location of Coq12 in *S. pombe*.** Notes: The enzymes involved in CoQ synthesis form a complex in *S. cerevisiae* (13) and this study. This figure illustrates the arrangement of CoQ biosynthetic and related proteins in the fission yeast and modified from the figure reported by Kawamukai (8). Proteins in the figure are not proportional to the actual molecular sizes. Some of the structure of Coq ortholog proteins are shown: *Aeropyrum pernix* UbiA (PDB entity: 4OD5) is shown as a Coq2/Ppt1 homolog, *E. coli* UbiG (PDB entity: 4KDC) is shown as a Coq3 homolog, Alr8543 from *Nostoc sp*. PCC7120 (PDB entity: 6E12) is shown as a Coq4 homolog, homologous proteins *S. cerevisiae* Coq5 (PDB entity: 4OBX) is shown as a Coq5 homolog, *E. coli* UbiI (PDB entity: 4K22) is shown as a Coq6 homolog, human ADCK3 (PDB entity: 4PED) is shown as a Coq8 homolog, human COQ7 and COQ9 complex (PDB entity: 7SSP) is shown as Coq7 and Coq9 homolog, and HpaC from *Thermus thermophilus* HB8 (PDB entity: 2ECR) is shown as a Coq12 homolog. Dps1+Dlp1 form heterotetrametric structure and it is separated from the main complex. A nonaprenyl diphosphate synthase (NDS) from *Rhodobacter capsulatus* (PDB entity: 3MZV) is shown as a Dps1 homolog. Coq2/Ppt1 spans the inner membrane. In *S. cerevisiae*, the positions of the other proteins have not been defined experimentally, although Coq4 seems to be in the center, and Coq7:Coq9 and Coq8 seem to be located at the edge of the complex. The PDB files were opened and edited by UCSF ChimeraX.

**Figure S10:**
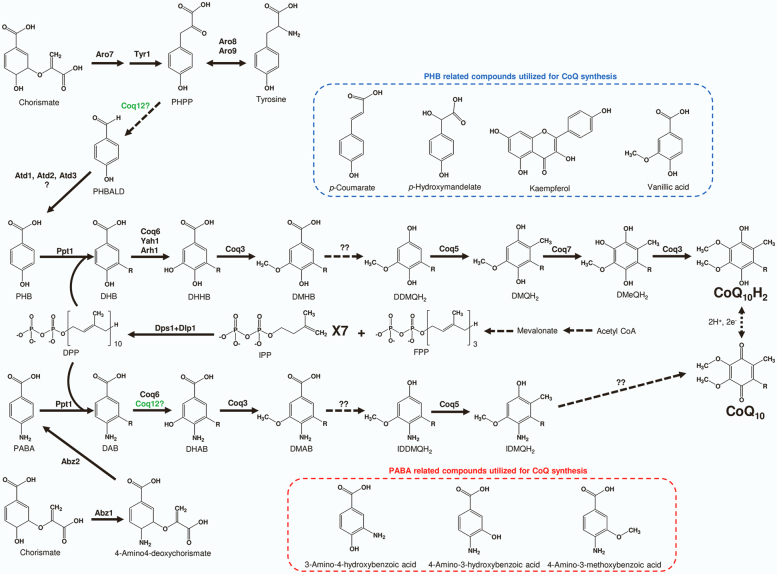
**Putative CoQ biosynthetic pathways in the yeast *S. pombe*.** Predicted Coq12 function and related aromatic compounds are shown. Coq12 is thought to be involved in an unknown metabolic process upstream of PHB. Coq12 may have a secondary role in Coq6 mediated hydroxylation step of PABA pathway. *p*-hydroxybenzoic acid (PHB), *p*-hydroxybenzaldehyde (PHBLD), *p*-coumarate, *p*-hydroxyphenylpyruvate (PHPP), Kaempferol, Vanillic acid, DL-*p*-hydroxymandelate, 3-amino-4-hydroxybenzoic acid, 4-amino-3-hydroxybenzoic acid, or 4-amino-3-methoxybenzoic acid restored CoQ_10_ level in *coq12* disruptant.

Synthesis of PHB in *S. cerevisiae* is known to be catalyzed by Hfd1 (24) but not proven in any other organisms. *S. pombe* contains three *HFD1* homologs, *atd1*, *atd2*, and *atd3*. We presumed that *atd1* is primarily involved in PHB synthesis based on our preliminary data. Deletion of *coq12* lowered the amount of the Coq4 protein, suggesting the *coq12* affects the protein complex comprised of Coq proteins. Our preliminary results suggest that Coq proteins form a complex in *S. pombe*, as has been well characterized in *S. cerevisiae* (13), but we have not yet confirmed the size of the protein complex in *S. pombe* (Fig. S9). The observation that PHB stabilizes the CoQ synthome in *S. cerevisiae* (13) supports the idea that Coq12 is involved in PHB synthesis. VA (3-methoxy PHB) restored the slow growth of the Δ*coq6* and Δ*coq12* strains, also supporting the involvement of Coq12 in the PHB pathway. Together, our results support a putative CoQ biosynthetic pathway in *S. pombe* (Fig. S10).

We think the primary role of Coq12 involves the PHB synthesis, but we also thought about the second role of Coq12 in the Coq6-mediated hydroxylation step (Fig. S8). This idea is based on the observation that addition of PABA did not restore CoQ content in Δ*coq12* strains, but hydroxylated PABA slightly restored the CoQ content. If Coq12 involves the hydroxylation step of PABA for utilization of PABA, it accounts for these observations.

We showed that purified Coq12 from *S. pombe* possesses NAD^+^-reducing activity using ethanol-extracted samples from *S. pombe*. There is clearly an unidentified substrate in *S. pombe*, other than PHBALC, because it does not react with purified Coq12 as a substrate. We added many possible products to the medium of growing Δ*coq12* cells, but clear restoration was only observed following addition of PHBALD, PHB, and VA. We believe that the substrate of Coq12 is an unknown compound that is converted to PHBALD. We also think that another protein must be necessary for full activity of Coq12 because purified Coq12 protein expressed in *E. coli* did not show NAD^+^-reducing activity, even though it was incubated with the as-yet unidentified substrate taken from *S. pombe*. We explored such a protein by mass spectrometry (MS)–based proteomic analysis using purified Coq12 protein from *S. pombe*. The Isa1, Isa2, and Mas2 proteins detected in the two separate samples from whole-cell and mitochondrial extracts are good candidates for a partner protein of Coq12 (Table S3). However, because these proteins are encoded by essential genes, we could not directly test the involvement of CoQ synthesis by constructing gene deletion strains. Among other mitochondrial proteins copurified with Coq12 (Table S3), we did not find corresponding gene deletion strains that affect CoQ synthesis, excluding CoQ biosynthetic genes such as *coq5*, *coq7*, and *coq9*. This may suggest that an essential gene is involved in CoQ synthesis, or other Coq proteins may work together with Coq12. Because Coq12 was copurified with Coq5, Coq7, and Coq9, and loss of functional Coq12 lowered the amount of Coq4 protein, it appears that Coq12 is a component of the Coq protein complex in *S. pombe*. More work is necessary to identify the substrate of Coq12 and the partner protein that functions together with Coq12.

In conclusion, we identified Coq11 and the novel protein Coq12, which are required for full CoQ synthesis in *S. pombe*. Both are mitochondrial proteins closely associated with known CoQ biosynthetic proteins. Coq12 functions as an NAD^+^ reductase enzyme with an as-yet unidentified partner in PHBALD or PHB synthesis. Because the PHB synthetic pathway is only poorly understood in eukaryotes, our work should pave the way for understanding the pathway leading to CoQ synthesis.

## Experimental procedures

### Yeast strains, *E. coli* strains, and culture media

Yeasts and *E. coli* strains used in this study are listed in Table S1. Standard yeast culture media and genetic methods were as described previously (46). *S. pombe* strains were grown in complete YES medium comprising 0.5% OXOID yeast extract (w/v), 3% glucose (w/v), and 225 mg/l each of adenine sulfate, leucine, uracil, histidine, and lysine hydrochloride. Nonfermentable carbon source medium (YEGES) was prepared by adding 2% glycerol (w/v) and 1% ethanol (w/v) instead of 3% glucose (w/v) to YES medium. For synthetic medium, Pombe minimal (PM) medium with 75 mg/l uracil and/or 75 mg/l leucine was used as necessary. WT cells or *coq* gene disruptants transformed with plasmid vectors were selected on PMU (PM containing uracil but lacking leucine) containing 10 μM thiamine and streaked onto the same media. Cultivation conditions using PM base media were described in each of the figure legends. *E. coli* cells were grown in complete LB medium comprising 0.5% yeast extract (w/v), 1% NaCl (w/v), and 1% HIPOLYPEPTON S (w/v).

### Construction of the disruptants

The *coq11* and *coq12* genes on the chromosomes were disrupted by a standard PCR-based gene disruption method as previously described (47). The 1.6 kb *kanMX6* module was amplified with flanking sequences corresponding to the target genes. Resistant colonies were selected on YES plates containing 100 mg/l G418, and disruption of *coq11* or *coq12* was verified by colony PCR. DNA fragments of 400 to 600 bp corresponding to the 5′ or 3′ regions of *coq11* gene or *coq12* gene were amplified by PCR using the coq11-d-W/SPAC1071.11del-A and coq11-d-X/SPAC1071.11del-B or coq11-d-Y/SPAC1071.11del-C and coq11-d-Z/SPAC1071.11del-D primer pairs (Table S2). For each gene, the amplified fragments were fused to the ends of the *kanMX6* module by PCR. The WT PR110 strain (Table S1) was transformed with the resulting *coq11::kanMX6* and *coq12(SPAC1071.11)::kanMX6* fragments to derive the disruptants. To confirm the chromosomal deletion of the *coq* genes, PCR was performed using the nb2, coq11-d-W, coq11-d-Z, coq11-d-chk1, and coq11-d-chk2 for coq11; nb2, SPAC1071.11del-A, SPAC1071.11del-D, SPAC1071.11del-check-1, and SPAC1071.11del-check-2 for *coq12* gene (Table S2); the resulting deletion strains were designated RYP26 (Δ*coq11*) and IN1 (Δ*coq12*), respectively.

### Plasmid construction

The plasmids were constructed as previous study (10). Primers used for plasmid construction are listed in Table S1. *S. pombe coq11* and *coq12* genes were PCR amplified using primers containing restriction sites, digested using restriction endonucleases, and then inserted into the appropriate sites of the pREP1 vector by ligation. pREP1-coq11 was constructed by inserting the PCR product amplified using the coq11(NdeI)-F and coq11(SalI)-R primers into the NdeI and SalI sites of pREP1. pREP1-coq12 was constructed similarly using SPAC1071.11(SalI)-F, SPAC1071.11(SmaI)-R. To examine the cellular localization of Coq12, GFP fusion was generated by inserting *coq12* into the pSLF172L-GFPS65A vector (48, 49). pSLF172L-coq12GFP was constructed by inserting the PCR product amplified using the SPAC1071.11GFP(XhoI)-F and SPAC1071.11-STOPGFP(BglII)-R primers into the XhoI and BglII sites of pSLF172L-GFPS65A. For protein purification using *S. pombe*, 8xHis tag fused Coq12 at C terminus was generated by inserting *coq12-8xHis* into the pREP1 vector. pREP1-coq12-8xHis was constructed by inserting the PCR product amplified using the SPAC1071.11(SalI)-F and coq12(-STOP) (NotI) 8xHis (SmaI) R primers into the SalI and SmaI sites of pREP1. For protein purification using *E. coli*, 6xHis tag fused Coq12 at N terminus was generated by inserting *coq12* into a Qiagen vector pQE-31. pQE-31-coq12 was constructed by inserting the PCR product amplified using the spac1071.11-SalI-F-2 and spac1071.11-HindIII-R. The insert genes were verified by DNA sequencing using appropriate primes for sequencing. The plasmids pREP1-coq4, pREP1-coq5, pREP1-coq8, and pREP1-coq9 were described previously (10). pREP1-Eco_ubiC was described previously (34). pREP1 vector containing thiamine-repressible *nmt1* promoter (50) was used to overexpress *coq4*, *coq5*, *coq8*, *coq11*, *coq12*, *coq12-8xHis*, and *ubiC*. The pREP41 vector containing the relatively weak promoter (*nmt41*) derived from the *nmt1* promoter of *S. pombe* (51) was used to overexpress the *ppt1* gene (28).

### CoQ extraction and measurement

The preculture of *S. pombe* cells was inoculated into a larger volume of medium and grown for the indicated time. Cell number was measured using a Sysmex CDA-1000B cell counter (Sysmex) or a CellDrop FL (DeNovix). Absorbance values were measured using a Shimadzu UVmini-1240 spectrophotometer (Shimadzu Corporation) or an Eppendorf BioSpectrometer kinetic (Eppendorf AG). At the indicated times, cells were harvested, and CoQ was extracted as described previously (10). The CoQ crude extract was analyzed by normal-phase TLC with authentic CoQ_6_ or CoQ_10_ standards. Normal-phase TLC was conducted on a Kieselgel 60 F_254_ plate (Merck Millipore) and developed with benzene. The plate was viewed under UV illumination, the CoQ band was collected, and samples were extracted with hexane/isopropanol (1:1, v/v). Samples were then dried and solubilized in ethanol. Purified CoQ was subjected to high-performance liquid chromatography on a Shimadzu HPLC Class *VP* series instrument (Shimadzu Corporation) equipped with a reversed-phase YMC-Pack ODS-A column (A-312-3 AA12S03-1506PT, 150 × 6 mm, internal diameter 3 μm, 120A, YMC). Ethanol was used as the mobile phase at a flow rate of 1.0 ml/min, and detection of CoQ was performed by monitoring absorption at 275 nm.

### Mitochondrial staining and fluorescence microscopy

Mitochondria were stained using Invitrogen MitoTracker Red FM dye (Thermo Fisher Scientific, Inc). The cells were suspended in PMU medium, and MitoTracker Red FM was added to a final concentration of 50 nM. After incubation at room temperature for 15 min, the cells were visualized at 1000× magnification using a BX51 fluorescent microscope (Olympus). The fluorescence of GFP^S65A^ was observed at an excitation wavelength of 485 nm. Fluorescent images were obtained using a digital camera (DP74; Olympus) connected to the microscope. Final merging of images was done using Adobe Photoshop 2022 software.

### Purification of His-tagged Coq12 from *E. coli* and *S. pombe*

For the purification of 6xHis-Coq12, *E. coli* BL21 harboring pQE-31-coq12 was grown in 500 ml of LB medium containing 100 μg/ml ampicillin (initial absorbance is approximately 0.02 at 600 nm) and incubated at 37 °C for about 3 h (absorbance at 600 nm = approximately 0.5). Cell cultures were once cooled down on ice, and then IPTG was added to make final concentration of 0.2 mM and cultivated for 24 h at 18 °C at 140 rpm. The IPTG-induced *E. coli* BL21/pQE-31-coq12 cells were collected in a high-speed cooling centrifuge (3500 rpm, 10 min, 4 °C) and washed with 20 ml of buffer A (50 mM sodium phosphate, 500 mM ammonium sulfate, pH 7.4). The cell solution was centrifuged and washed (3500 rpm, 10 min, 4 °C). The cell pellet was resuspended in 20 ml of buffer A containing 1 mM PMSF at a final concentration of 1 mM and then sonicated on ice using an ultrasonic sonicator machine VP-050N (TAITEC) (30 s on, 30 s off, at least ten cycles, PWM 65%). The supernatant was collected by centrifugation (10,000*g*, 15 min, 4 °C) and filtered through a 0.2 μm syringe filter to obtain the cell-free extract. At 4 °C, His-tag purification beads were equilibrated with 5 ml of binding buffer (buffer A + 10 mM imidazole [pH 7.4]), and the resulting cell-free extract was passed through a column. After washing with 5 ml of wash buffer (buffer A + 70 mM imidazole [pH 7.4]), the eluted fraction was collected in 5 ml of elution buffer (buffer A + 300 mM imidazole [pH 7.4]) and used as the purified enzyme. For the purification of Coq12-8xHis from *S. pombe*, the WT strain (PR110) harboring pREP1-coq12-8xHis was grown in PMU medium supplemented with 0.15 μM thiamine. About 5 ml of the preculture was inoculated into 500 ml of PMU + 0.15 μM thiamine medium in 1 l flask to an absorbance of 0.2 at 600 nm. About 500 ml × two sets of yeast culture were collected (absorbance at 600 nm reached 5580 units/1 l and incubated with 1 mM final concentration of buffer A containing 1 mM PMSF was used for glass bead disruption using a Multi-beads Shocker MB1001 (Yasui Kikai) (2500 rpm, on 60 s off 60 s × 6) twice. Here, half the volume of the buffer was added for the second disruption, obtaining a total of approximately 30 ml of crude extract. About 2 ml crush tubes were prepared, each containing cells equivalent to an absorbance of 300 units at 600 nm. About 300 μl of 0.5 mm beads were added to each tube. The resulting extract was centrifuged in a high-speed cooling centrifuge (10,000*g*, 15 min, 4 °C), and the supernatant was collected and filtered through a 0.2 μm syringe filter. The cell-free extract was subjected to His-tag purification at 4 °C similar as in the case of *E. coli* experiment. Electrophoresis (SDS-PAGE) on a 13% SDS-polyacrylamide gel was performed to confirm purification for both *E. coli* and *S. pombe* experiments.

### Measurement of NADH

*S. pombe* WT strain (PR110) and the Δ*coq12* cells were inoculated to adjust an absorbance of approximately 0.2 at 600 nm (approximately 1 × 10^6^ cells/ml), and 330 ml of YES medium containing the cells were incubated for 1 day and collected. After washing three times with deionized pure water, the cells were concentrated to an absorbance of 60 per ml at 600 nm and incubated for 15 min at 80 °C with 100% ethanol for metabolite extraction. These were vacuum dried, resuspended in pure water, and filtered for activity assays using purified Coq12-8xHis protein. The reaction condition was followed by the method measuring benzyl alcohol dehydrogenase activity (52). The composition of the reaction solution was as follows (83 mM sodium phosphate, 40 mM KCl, 2 mM NAD^+^, yeast extract, 0.25 mM EDTA buffer, pH 7.3, and 1 μg of purified enzyme). About 100 μl of the extract was used for the analysis. The amount of NADH produced with and without addition of substrate was examined; the reaction was carried out at 20 °C. In the experiments, changes in absorbance at 339 nm were measured by a Shimadzu UVmini-1240 spectrophotometer (Shimadzu Corporation).

### Antibodies

To immunochemically detect CoQ biosynthetic proteins, rabbit polyclonal antisera were prepared by Sigma–Aldrich by injecting rabbits with specific peptides of Coq proteins (53). The specificity of antisera against each of the CoQ biosynthetic proteins (Dlp1, diluted 1:1000; Coq4, diluted 1:500) was assessed by Western blot analysis. Preparation of cell lysates and detection of CoQ biosynthetic proteins by immunoblotting *S. pombe* cell lysates were performed as described previously (28). *S. pombe* WT (PR110) cells were inoculated into 55 ml YES main cultures with or without Bz (initial cell density ∼1 × 10^5^ cells/ml) and incubated with rotation at 30 °C for 2 days and then harvested. Lysate proteins were separated by SDS-PAGE, after which Western blot analysis was performed using an ECL detection system (GE Healthcare) or Amersham ECL Western Blotting Detection Reagent RPN2106 (Cytiva). Rabbit polyclonal antibodies against the PSTAIRE peptide (Cdc2, diluted 1:1000) were purchased from Santa Cruz Biotechnology. Horseradish peroxidase–conjugated anti-rabbit immunoglobulin G antibody (Promega) was used as secondary antibody (diluted 1:2000). These antibodies were dissolved in Can Get Signal immunostain (TOYOBO), an immunoreaction enhancer solution. For quantification of protein bands, ImageJ (https://imagej.nih.gov/ij/download.html) was used.

### Proteomic analysis of Coq12-interacting proteins

*S. pombe* WT (PR110) cells harboring the empty vector pREP1 or pREP1-coq12-8xHis were cultured in 2 l of PMU medium containing 0.15 μM thiamine for 1 day. Whole cells and mitochondrial fractions (28, 54) were obtained, and the respective soluble fractions were filtered and purified with nickel–nitrilotriacetic acid agarose and eluted with 50 mM sodium phosphate, 500 mM ammonium sulfate, 300 mM imidazole (pH 7.4) and eluted. Samples were mixed with elution buffer in the ultrafiltration units and centrifuged at 10,000*g* until less than 50 μl of sample remains above the filter. About 500 μl of 50 mM Tris–HCl (pH 8.0) buffer was added to the ultrafiltration units, and the centrifugation was repeated until sample solution became less than 50 μl. This step was repeated twice. About 12.5 μl of 9 M urea was added to the concentrate collected from the ultrafiltration units and were evaporated in a SpeedVac vacuum concentrator. The residues were solubilized in 15 μl of 50 mM Tris–HCl (pH 8.0) followed by reduction in 10 mM DTT at 37 °C for 30 min and alkylation in 25 mM iodoacetamide at room temperature for 20 min in the dark. After reducing the urea concentration to 2 M with 50 mM Tris–HCl (pH 8.0), the proteins were digested with 500 ng of trypsin at 37 °C overnight. Peptide samples were desalted using C18 stop-and-go-extraction tips (StageTips) and evaporated in the SpeedVac vacuum concentrator (55). The dried peptides were dissolved in 2% acetonitrile containing 0.1% formic acid. The peptide sample was transferred to an MS vial (ProteoSave vial [catalog no.: 11-19-1021-10]; AMR, Inc), and one-fifth volume of peptide samples were subjected to nanoLC–MS/MS using a Q Exactive system (Thermo Fisher Scientific, Inc) equipped with an autosampler. The loaded peptides were separated on a 75 μm inner diameter × 150-mm C18 reversed-phase column (Nikkyo Technos) using UltiMate 3000 RSLCnano (Thermo Fisher Scientific, Inc). The mobile phase was composed of 0.1% formic acid (solution A) and 0.1% formic acid in 90% acetonitrile (solution B). For the proteomics analysis, a flow rate of 350 nl/min of 5% solution B for 5 min, 5 to 40% solution B for 115 min, 40 to 95% solution B for 0.1 min, 95% solution B for 10 min, 95 to 5% solution B for 0.1 min, and 5% solution B was used. The mass spectrometer was operated in data-dependent acquisition mode with a top ten MS/MS method. MS1 spectra were measured with a resolution of 70,000, an automatic gain control target of 1e6, and a mass range from 300 to 1500 *m/z*. MS/MS spectra were acquired at a resolution of 17,500, an automatic gain control target of 5e4, an isolation window of 2.0 *m/z*, a maximum injection time of 60 ms, and a normalized collision energy of 27. Dynamic exclusion was set to 20 s. Peptides and proteins were identified through automated database searching using MaxQuant software (version 1.6.17.0) (Max Planck Institute of Biochemistry) in the label-free quantitation mode against the *S. pombe* database from UniProtKB/Swiss-Prot release 2020/01 with a strict trypsin/P specificity allowing for up to two missed cleavages. Carbamidomethyl (C) was set as a fixed modification. Oxidation (M) and acetylation (Protein N-term) were allowed as variable modifications. For statistical analysis of MaxQuant output, Perseus (Max Planck Institute of Biochemistry) framework was used (56). For Coq12 interaction analysis, a group of Coq12-8xHis purified proteins from each of whole and mitochondria were compared with a control strain harboring empty vector. First, the software Perseus was started, data were loaded, continuous variable were preprocessed, annotation information was added, volcano plots were drawn, and the results of the analysis were saved. From the plots, the proteins that were abundant in the Coq12 group were considered as candidate of interacting proteins. Data were analyzed at Medical ProteoScope Co., Ltd.

### Data and statistical analyses

Data from control and target samples were compared using the two-sample *t* test in Microsoft Excel, and *p* values <0.05 were considered statistically significant.

## Data availability

The data published in this article would be available upon request to the corresponding author.

## Supporting information

This article contains the supporting information (Table S1; 10, 29, 31, 47, 57).

## Conflict of interest

The authors declare that they have no conflicts of interest with the contents of this article.
