# Supplementary material for: Identification of novel coenzyme Q10 biosynthetic proteins Coq11 and Coq12 in Schizosaccharomyces pombe
Source: J Biol Chem. 2023 May 6;299(6):104797. doi: 10.1016/j.jbc.2023.104797 (PMC10279924; doi:10.1016/j.jbc.2023.104797)
Supplement: Table S1 [file mmc1.pdf]

Table S1 Microorganisms strains used in this study

Strain	Genotype	Origin
S. pombe		
PR110	*h^+ leu1-32 ura4-D18*	P. Russell
LJ1030	*h^+ leu1-32 ura4-D18 dps1::kanMX6*	[31]
RM19	*h^+ leu1-32 ura4-D18 dlp1::kanMX6*	[47]
LA1	*h^+ ade6-M210 leu1-32 ura4-D18 dps1::kanMX6 dlp1::ura4::ADE2*	[31]
KH2 (OG1)	*h^+ leu1-32 ura4-D18 ppt1::kanMX6*	[10]
KH3 (RM2)	*h^+ leu1-32 ura4-D18 coq3::kanMX6*	[47]
KH4 (LV974)	*h^+ leu1-32 ura4-D18 coq4::kanMX6*	[10]
KH5 (RM7)	*h^+ leu1-32 ura4-D18 coq5::kanMX6*	[10]
KH6 (PC976)	*h^+ leu1-32 ura4-D18 coq6::kanMX6*	[10]
KH7 (RM1)	*h^+ leu1-32 ura4-D18 coq7::kanMX6*	[47]
KH8 (OG2)	*h^+ leu1-32 ura4-D18 coq8::kanMX6*	[10]
KH9 (KTC9)	*h^+ leu1-32 ura4-D18 coq9::kanMX6*	[10]
CZ48	*h^+ leu1-32 ura4-D18 coq10::kanMX6*	[57]
RYP26	*h^+ leu1-32 ura4-D18 coq11::kanMX6*	This study
IN1	*h^+ leu1-32 ura4-D18 coq12::kanMX6*	This study
Bioneer disruptants (Ver. 4)	*h^+ ade6-M210(216) leu1-32 ura4-D18 geneX::kanMX4*	[29]
E. coli		
DH5α	F^- Φ 80*lac*Z Δ M15 Δ (*lac*ZYA-*arg*F) U169 *rec*A1 *end*A1 *hsd*R17 (rK^-, mK^+) *pho*A *sup*E44 λ– *thi*-1 *gyr*A96 *rel*A1	Lab stock
BL21	F^- *ompT hsdS*$_B$ (r_B^- m_B^-) *gal dcm*	Lab stock
