# Supplementary material for: Identification of novel coenzyme Q10 biosynthetic proteins Coq11 and Coq12 in Schizosaccharomyces pombe
Source: J Biol Chem. 2023 May 6;299(6):104797. doi: 10.1016/j.jbc.2023.104797 (PMC10279924; doi:10.1016/j.jbc.2023.104797)
Supplement: Table S2 [file mmc2.pdf]

Table S2 Primers used in this study

Name	Sequence
pFA6a-F	5'-TCGTACGCTGCAGGTCGACG-3'
pFA6a-R	5'-CATCGATGAATTCGAGCTCG-3'
pQE-31-F	5'-GTGAGCGGATAACAATTTCA-3'
pQE-31-R	5'-CTAGCTTGGATTCTCACC-3'
Nb2	5'-GTTTAAACGAGCTCGAATTC-3'
nmt1pro-seq_Fw	5'-AAAACCGGATAATGGACCTG-3'
nmt1term-Seq_Rv	5'-AAACAAAATCGTAATATGCAGC-3'
coq11(NdeI)-F	5'-CGCCATATGATGAAAATTGTTGTTTTAGG-3'
coq11(SalI)-R	5'-CGCGTCGACTCATAGAGATTTTTGCTTAA-3'
coq11-d-W	5'-GCGCTCTCATTTAAGACAAAC-3'
coq11-d-X	5'-GGGGATCCGTCGACCTGCAGCGTACGACAATTTGATATAGGCCAGCCC-3'
coq11-d-Y	5'-GTTTAAACGAGCTCGAATTCATCGATGCCAAATTTGCTTCCTGACTG-3'
coq11-d-Z	5'-GTAATCGCGAACAGGAACGC-3'
coq11-d-chk1	5'-CAGTGTCAAGGGTCCTGTGG-3'
coq11-d-chk2	5'-CTTCTCCTTCTGGTGAATGC-3'
SPAC1071.11(SalI)-F	5'-CGCGTCGACAATGTCAATCCGATTTACTC-3'
SPAC1071.11(SmaI)-R	5'-ATACCCGGGTTAACTTTCGAAAGAATCAATAA-3'
coq12(-STOP)(NotI)8xHis(SmaI)R	5'-ATACCCGGGTTAATGGTGGTGATGGTGATGATGATGGGCGGCCGCAC TTTCGAAAGAATCAATAA-3'
SPAC1071.11GFP(XhoI)-F	5'-CCGCTCGAGAATGTCAATCCGATTTACTCA-3'
SPAC1071.11-STOPGFP(BglII)-R	5'-CCGAGATCTCCACTTTCGAAAGAATCAATAA-3'
SPAC1071.11-seq-1	5'-CGTTCATCTTCTGTCTAGTT-3'
spac1071.11-SalI-F-2	5'-CGCGTCGACATGTCAATCCGATTTACTCA-3'
spac1071.11-HindIII-R	5'-ATAAAGCTTTTAACTTTCGAAAGAATCAA-3'
SPAC1071.11del-A	5'-CCACTACTCATCAGATTCGC-3'
SPAC1071.11del-B	5'-GGGGATCCGTCGACCTGCAGCGTACGACAAAATATGTAAACAATTATTAAG-3'
SPAC1071.11del-C	5'-GTTTAAACGAGCTCGAATTCATCGATTTCTTTTTTTGAGTTTGCTAC-3'
SPAC1071.11del-D	5'-GCTTTTAAAGGTATTAAGTTCGCCC-3'
SPAC1071.11del-check-1	5'-GCAGCACATCCCCGTTAAG-3'
SPAC1071.11del-check-2	5'-CTTATTATGGAGGAAGCGATGG-3'

Primers used for construction of plasmids and disruptants are listed. Underlines indicate restriction enzyme site.
