# Supplementary material for: Identification of novel coenzyme Q10 biosynthetic proteins Coq11 and Coq12 in Schizosaccharomyces pombe
Source: J Biol Chem. 2023 May 6;299(6):104797. doi: 10.1016/j.jbc.2023.104797 (PMC10279924; doi:10.1016/j.jbc.2023.104797)
Supplement: Table S3 [file mmc3.pdf]

Table S3 Proteins highly associated with Coq12

	Gene product	Function	Fraction	
			Whole cell	Mitochondria
1	Isa1	Iron-sulfur assembly protein 1	+	+
2	Isa2	Iron-sulfur assembly protein 2	+	+
3	Mas2	Probable mitochondrial-processing peptidase subunit alpha	+	+
	SPAC1071.11 (Coq12)	Uncharacterized protein C1071.11	+	+
4	Coq9	Ubiquinone biosynthesis protein Coq9, mitochondrial	+	
5	Pab2	Polyadenylate-binding protein 2	+	
6	Leu3	2-isopropylmalate synthase	+	
7	Utp10	U3 small nucleolar RNA-associated protein 10	+	
8	Coq7	5-demethoxyubiquinone hydroxylase, mitochondrial	+	
9	Coq5	2-methoxy-6-polyprenyl-1,4-benzoquinol methylase, mitochondrial	+	
10	Hsp60	Heat shock protein 60, mitochondrial	+	
11	Pus1	TruA family tRNA/ U2 snRNA pseudouridine synthase Lsp1	+	
12	Ppk6	Serine/threonine-protein kinase Ppk6	+	
13	SPBC2F12.10	54S ribosomal protein L35, mitochondrial		+
14	Kes1	Sterol transfer protein Kes1		+
15	Zta1	Probable quinone oxidoreductase		+
16	Hrd1	ERAD-associated E3 ubiquitin-protein ligase Hrd1		+
17	SPCC622.06c	Putative uncharacterized membrane protein C622.06c		+
18	Msp1	Mitochondrial dynamin family fusion GTPase Msp1		+
19	Bur6 (previous Dpb3)	Transcription regulator complex subunit Bur6		+
20	Glo3	ADP-ribosylation factor GTPase-activating protein Glo3		+
21	Mug161	CwfJ family protein, splicing factor		+
22	Sir1	Sulfite reductase [NADPH] subunit beta		+
23	SPCC4B3.11c	Mitochondrial [4Fe-4S] cluster transfer protein Fra3		+
24	Tsc2	Tuberin, GTPase activator Tsc2		+
