# Supplementary material for: Identification of novel coenzyme Q10 biosynthetic proteins Coq11 and Coq12 in Schizosaccharomyces pombe
Source: J Biol Chem. 2023 May 6;299(6):104797. doi: 10.1016/j.jbc.2023.104797 (PMC10279924; doi:10.1016/j.jbc.2023.104797)
Supplement: Table S4 [file mmc4.pdf]

Table S4 Coq proteins associated with Coq12 (Whole cell)

Coq protein	LFQ value (Coq12-vector)<br>*Average of two experiments
Coq5	304,740,500
Coq6	10,407,650
Coq7	26,168,100
Coq9	10,359,100
SPAC1071.11 (Coq12)	56,926,357,725
