# Supplementary material for: Identification of novel coenzyme Q10 biosynthetic proteins Coq11 and Coq12 in Schizosaccharomyces pombe
Source: J Biol Chem. 2023 May 6;299(6):104797. doi: 10.1016/j.jbc.2023.104797 (PMC10279924; doi:10.1016/j.jbc.2023.104797)
Supplement: Table S5 [file mmc5.pdf]

Table S5 Coq proteins associated with Coq12 (Mitochondria)

Coq protein	LFQ value (Coq12-vector)<br>*Average of two experiments
Coq3	8,319,000
Coq4	9,169,000
Coq5	912,310,400
Coq6	24,738,000
Coq7	69,449,100
Coq8 (Abc1)	55,923,250
Coq9	25,137,500
SPCC1840.09 (Coq11)	27,498,000
SPAC1071.11 (Coq12)	69,583,848,000
Atd1	61,944,750
